# Indices of airway resistance and reactance from impulse oscillometry correlate with aerosol particle emission in different age groups

**DOI:** 10.1038/s41598-024-55117-2

**Published:** 2024-02-26

**Authors:** Benedikt Schumm, Stephanie Bremer, Katharina Knödlseder, Martin Schönfelder, Rainer Hain, Luisa Semmler, Elke Lorenz, Henning Wackerhage, Christian J. Kähler, Rudolf Jörres

**Affiliations:** 1 Department of Aerospace Engineering, Institute of Fluid Mechanics and Aerodynamics, Universität der Bundeswehr München, 85577 Neubiberg, Germany; 2 Professorship of Exercise Biology, Department of Sport and Health Sciences, Technische Universität München, 80809 Munich, Germany; 3 Department of Neurology, Klinikum Rechts der Isar, Technische Universität München, 81675 Munich, Germany; 4 Klinik für Herz- und Kreislauferkrankungen, Deutsches Herzzentrum München, Technische Universität München, 80636 Munich, Germany; 5 Institute and Outpatient Clinic for Occupational, Social and Environmental Medicine, LMU Hospital, Comprehensive Pneumology Center Munich (CPC-M), Member of the German Center for Lung Research (DZL), Ludwig Maximilian University of Munich, Munich, Germany

**Keywords:** Ageing, Respiration, Infectious diseases

## Abstract

Airborne transmission of pathogens plays a major role in the spread of infectious diseases. Aerosol particle production from the lung is thought to occur in the peripheral airways. In the present study we investigated eighty lung-healthy subjects of two age groups (20–39, 60–76 years) at rest and during exercise whether lung function parameters indicative of peripheral airway function were correlated with individual differences in aerosol particle emission. Lung function comprised spirometry and impulse oscillometry during quiet breathing and an expiratory vital capacity manoeuvre, using resistance (R5) and reactance at 5 Hz (X5) as indicators potentially related to peripheral airway function. The association between emission at different ventilation rates relative to maximum ventilation and lung function was assessed by regression analysis. In multiple regression analyses including age group, only vital capacity manoeuvre R5 at 15% to 50% of end-expiratory vital capacity as well as quiet breathing X5 were independently linked to particle emission at 20% to 50% of maximum ventilation, in addition to age group. The fact that age as predictive factor was still significant, although to a lower degree, points towards further effects of age, potentially involving surface properties not accounted for by impulse oscillometry parameters.

## Introduction

Aerosol particles are endogenously generated in the respiratory tract^1,2^ and are vehicles for pathogens that cause airborne diseases^3^. In a previous study, we compared aerosol particle emission (particles/min) between older (67.4 ± 4.4 years) and younger (27.1 ± 4.9 years) lung-healthy women and men at rest and during graded exercise up to exhaustion^4^. We found that elderly subjects emitted ≈ twofold more particles/min during rest and exercise. This revealed that elevated aerosol particle emission is a feature of lung ageing that is currently not recognised as such^5^. The mechanism that increases aerosol particle emission in older lungs is, however, unknown.

Aerosol particle generation in the lung probably occurs by collapse and subsequent re-opening of small airways during the breathing cycle^6^. Thus alterations in small airway function and morphology are associated with changes in aerosol particle generation. Since the morphology and function especially of the peripheral airways changes during normal aging^7,8^, these changes could be the cause for the elevated aerosol particle emission in elderly subjects when compared to younger subjects. However, to date, it is unclear which of the aging-induced changes of the respiratory system causes increased aerosol emission.

In general, the maximum expiratory flow rates at low lung volumes are indicators of such alterations of the peripheral airways^7^, and this is consistent with the observation that obstructive airway diseases typically start with impairments in small airway function and morphology^9^. The maximum expiratory flow rates are, however, determined during forced expiration which does not necessarily reflect the mechanical conditions encountered during rest and at non-maximum ventilation. For this, other methods have been proposed, such as impulse oscillometry, which provides indices that depend on a variety of mechanical properties of the lung including those of central and peripheral airways. These indices are commonly expressed as resistance and reactance at specific frequencies and assessed during resting ventilation or specific breathing manoeuvres^10,11^.

In one of our previous studies^12^, we described aerosol particle concentration and emission during rest and exercise in younger, healthy subjects, and in a subsequent study we extended this by including older subjects^4^. However, these earlier studies did not identify the mechanisms underlying the increase in aerosol particle emission e.g., during exercise or in elderly subjects. The aim of the present study was therefore to combine data of our published study^4^ with additional lung function data including impulse oscillometry data to answer the following research question: Do lung function data predict aerosol particle emission at rest versus during exercise, and in young versus elderly subjects?

## Methods

### Study participants

All study participants were required to be healthy with respect to their clinical history and to be free of respiratory infections within 2 weeks prior to the tests. Additionally, subjects had to be never-smokers or ex-smokers since at least 6 months. We recruited younger subjects aged 20–40 years and elderly subjects aged 60–75 years. The study was approved by the Ethical Commission of the Technical University of Munich (239/21S-SR), and all participants gave their written, informed consent.

### Study protocol

Prior to inclusion, subjects were tested for SARS-CoV-2 by antigen testing. If negative, eligibility was determined based on medical examination, including the determination of resting ECG, blood pressure and clinical history. All participants were required to have no infection, respiratory symptoms, history of respiratory or cardiac disease or impairments preventing the participation in the exercise test. We measured height and weight and performed a spirometry as well as impulse oscillometry, followed by a graded exercise test on a cycle ergometer until exhaustion, during which ventilation and aerosol particle concentration were measured simultaneously. Lung function and exercise tests were performed within one hour, and all measurements were carried out in accordance with relevant institutional guidelines and regulations.

### Spirometry

Spirometry (Spiroscout, Ganshorn Medizin Electronic GmbH, Niederlauer, Germany) provided values of forced expiratory volume in 1 s (FEV_1_), forced vital capacity (FVC) and maximum flow rates at 75%, 50% and 25% of the expired volume (MEF75, MEF50, MEF25). The assessment followed current recommendations^13^ with regard to performance and quality control; measurements were performed at least in duplicate and, if needed, repeated until consistent values were obtained. Predicted values were taken from the equations of the Global Lung Function Initiative (GLI)^14^.

### Impulse oscillometry

**Figure 1:**
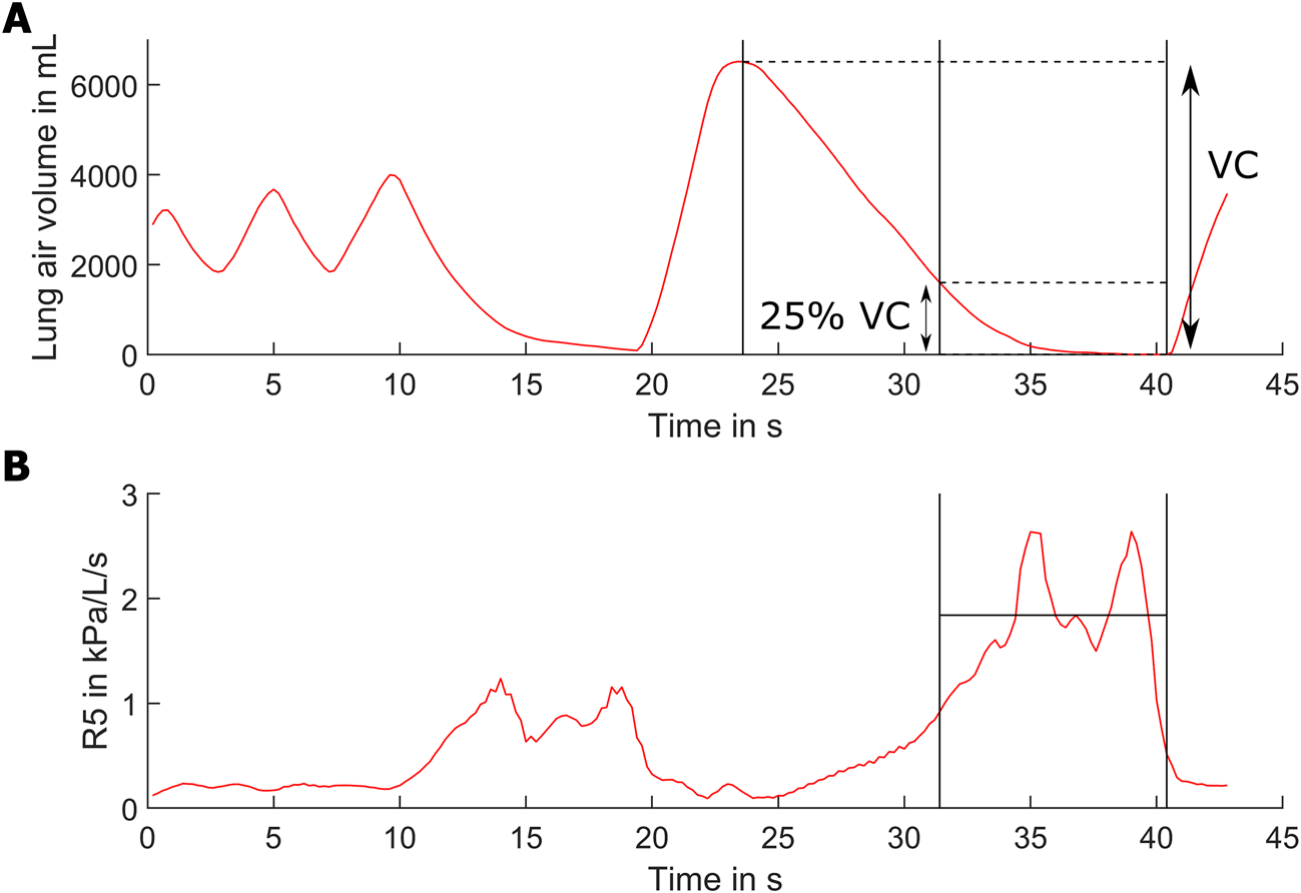
Time course of volume (**A**) and the resistance at 5 Hz (**B**) during the impulse oscillometry vital capacity maneuver. Legend: The vertical lines indicate the timespan of the final 25% of vital capacity as an example of the averaging window. As shown in (**B**), the average value of R5 over this volume was defined as R5ex25. In analogy, values for 5%, 15%, 40% and 50% were computed, thus covering different ranges of lung volume.

Measurements were performed using the device MS-IOS Digital (Jaeger, Vyaire, Höchberg, Germany) imposing 5 impulses per second, first during quiet breathing over 1 min in duplicate. Fourier analysis of these pulses was used to determine the resistance values at 5 Hz and 20 Hz (R5 and R20, respectively), as well as reactance (X5) at 5 Hz, averaged over 60 s. The selection of the two frequencies followed common practice and was motivated by the fact that 5 Hz and 20 Hz cover a frequency range that is considered to be informative for airway function^10,11^ including properties that could be relevant for particle generation. The quiet breathing was followed by a vital capacity manoeuvre to determine the dependence of resistance from lung volume. After deep inspiration, a deep expiration was performed, during which resistance was monitored; an example of the manoeuvre is illustrated in Fig. 1A. As can be seen, flow rate and duration of the manoeuvre were chosen to be low enough to ensure quasistatic conditions and avoid dynamic effects. This manoeuvre was also done at least in duplicate, until reproducible tracings were obtained. For data analysis, the average resistance at 5 Hz was computed over defined end-expiratory volumes, which were chosen as 5%, 15%, 25%, 40%, 50% of vital capacity. This is illustrated in Fig. 1A for 25%, and respective values are termed R5exXX, with XX designating the percentage; a tracing of R5 is shown in Fig. 1B.

### Exercise testing

All participants performed a cardiopulmonary exercise test on a stationary bicycle ergometer (Ergoline Ergoselect 200, Lode, Groningen, Netherlands) during which ventilation parameters were measured as described previously^4^.

### Aerosol particle measurements

An optical particle counter (Promo 3000 particle spectrometer combined with a Welas 2300 sensor; Palas GmbH, Karlsruhe, Germany) was used to measure aerosol particle concentration and particle size at rest and during exercise. A full description of the experimental setup is given in Ref.^4^.

### Data analysis

For data description, mean values, standard deviations (SD) and ranges were chosen. All data regarding exercise test and aerosol particle measurements were extracted from the raw files and processed with MATLAB (R2021b, The MathWorks Inc, Natick, MA, USA); mean values were calculated for each time interval of the exercise test. Data regarding the impulse oscillometry vital capacity manoeuvre were also taken from the raw data files, while impulse oscillometry data obtained during quiet breathing and spirometric data were those computed by the respective devices. For each individual, the relationship between ventilation and logarithmically transformed values of aerosol particle emission was described via linear regression analysis. Based on this, emission values at defined percentages of individual maximum ventilation (15% to 50%) were derived. The relationship between aerosol particle emission at defined relative ventilation rates and R5exXX or X5 values resulting from multiple regression analyses was visualized in a heatmap indicating the significance levels for the respective regression coefficients (Table S1). Statistical comparisons were performed by unpaired t-tests, and associations were analysed by multiple regression analysis as well as Pearson’s and Spearman correlation coefficients. Significance was assumed for p < 0.05. All statistical analyses were performed with the package SPSS version 28 (IBM, Armonk, NY, USA).

## Results

### Study population and lung function data

The subjects of this study were the same as in Schumm et al.^4^, i.e., 40 women and men aged 20–39 years and 40 women and men aged 60–76 years, respectively (Table 1). The person aged 76 years (above our inclusion criteria) was 75 at recruitment. Several subjects had FEV_1_ (n = 3) or FEV_1_/FVC (n = 16) values below the lower limit of normal (LLN)^14^, without a history of respiratory disease or symptoms. The two age groups were significantly different for FEV_1_, FVC, FVC % predicted and z-Score (p < 0.05, each).

**Table 1:** Participant characteristics.

	Young (n = 40)	Old (n = 40)	p value
Age (years)	27.1 ± 4.9 (20 to 39)	67.4 ± 4.4 (60–76)	–
Sex (F/M)	20/20 (50%/50%)	20/20 (50%/50%)	–
Height (cm)	175.0 ± 11.2 (151.3 to 193.0)	171.2 ± 8.3 (158.0 to 192.2)	0.085
BMI (kg/m^2^)	23.8 ± 3.3 (18.0 to 30.9)	25.1 ± 3.3 (18.5 to 33.3)	0.075
Smoking status (ex/never)	11/29 (27.5%/72.5%)	18/22 (45.0%/55%)	0.106
FEV_1_ (L)	4.3 ± 1.0 (1.9 to 6.1)	3.0 ± 0.7 (1.7 to 4.2)	< 0.001
FEV_1_ (% pred)	103.0 ± 14.2 (67.8 to 143.1)	105.1 ± 16.1 (56.9 to 138.2)	0.624
FEV_1_ z-Score	0.25 ± 1.15 (− 2.70 to + 3.75)	0.34 ± 1.03 (− 2.50 to + 2.48)	0.692
FVC (L)	5.3 ± 1.4 (3.3 to 7.5)	4.3 ± 0.9 (2.5 to 6.2)	< 0.001
FVC (% pred)	103.7 ± 12.4 (81.8 to 142.9)	106.3 ± 14.7 (71.6 to 138.2)	< 0.001
FVC z-Score	0.56 ± 1.07 (− 1.15 to + 4.70)	1.12 ± 0.87 (− 1.38 to + 2.55)	0.005

Mean values ± standard deviation as well as range (in parentheses), or numbers and percentages (in parentheses) are shown.

*BMI* body mass index, *FEV*_*1*_ forced expiratory volume in one second, *FVC* forced vital capacity, predicted values based on *GLI* global lung function initiative.

All forced expiratory flow rates (Table 2) differed significantly (p < 0.001, each) between the younger and elderly subjects; if expressed as % predicted, MEF75 and MEF50 differed (p = 0.029 and p < 0.001) but not MEF25. In one subject these values were not available due to technical reasons. Neither at 5 Hz nor at 20 Hz the values of impulse oscillometry obtained during quiet breathing differed significantly between the younger and older group. In contrast the differences of these resistance values (R5-R20) did (p = 0.007). Impulse oscillometry values obtained with vital capacity manoeuvre also showed significant differences between the two age groups regarding R5ex50 and R5ex40.

**Table 2:** Parameters of flow volume curve and impulse oscillometry.

	Young (n = 40)	Elderly (n = 40)	p value
Flow volume curve			
MEF75 (L/s)	7.26 ± 1.97 (2.27 to 11.35)	5.28 ± 1.75 (1.80 to 10.72)	< 0.001
MEF50 (L/s)	4.94 ± 1.46 (1.63 to 9.26)	2.85 ± 1.29 (0.67 to 5.60)	< 0.001
MEF25 (L/s)	2.20 ± 0.88 (0.68 to 3.91)	0.64 ± 0.35 (0.13 to 1.52)	< 0.001
MEF75 (% pred)	0.97 ± 0.24 (0.39 to 1.58)	0.85 ± 0.27 (0.34 to 1.49)	0.029
MEF50 (% pred)	0.96 ± 0.25 (0.38 to 1.62)	0.73 ± 0.31 (0.20 to 1.45)	< 0.001
MEF25 (% pred)	1.09 ± 0.40 (0.34 to 2.04)	0.99 ± 0.55 (0.29 to 2.52)	0.367
Impulse oscillometry, quiet breathing			
R5 mean (kPa/L/s)	0.66 ± 0.31 (0.33 to 1.77)	0.76 ± 0.27 (0.32 to 1.43)	0.141
R20 mean (kPa/L/s)	0.45 ± 0.21 (0.20 to 1.31)	0.43 ± 0.14 (0.22 to 0.77)	0.708
R5-R20 mean (kPa/L/s)	0.21 ± 0.18 (-0.07 to 0.90)	0.33 ± 0.18 (0.05 to 0.81)	0.007
X5 mean (kPa/L/s)	− 0.09 ± 0.03 (− 0.17 to − 0.03)	− 0.09 ± 0.04 (− 0.25 to − 0.04)	0.573
Impulse oscillometry, vital capacity manoeuvre			
R5ex50 (kPa/L/s)	1.12 ± 0.61 (0.47 to 3.79)	1.39 ± 0.50 (0.38 to 2.70)	0.029
R5ex40 (kPa/L/s)	1.16 ± 0.64 (0.48 to 4.08)	1.43 ± 0.51 (0.39 to 2.73)	0.040
R5ex25 (kPa/L/s)	1.24 ± 0.73 (0.50 to 4.75)	1.50 ± 0.53 (0.40 to 2.82)	0.071
R5ex15 (kPa/L/s)	1.30 ± 0.80 (0.47 to 5.28)	1.56 ± 0.54 (0.42 to 2.74)	0.093
R5ex5 (kPa/L/s)	1.45 ± 1.05 (0.45 to 6.80)	1.55 ± 0.59 (0.44 to 2.95)	0.602

All data are mean values ± standard deviation as well as range (in parentheses).

### Exercise data

Maximum power and maximum ventilation rates as well as rates at 20% and 50% (Table 3) differed significantly between the age groups (p < 0.001, each) and between men and women (p < 0.001, each). The relationship between relative power and absolute ventilation rates is illustrated in Fig. S1. To achieve comparability between subjects ventilation was transformed into percentages of individual maximum ventilation.

**Table 3:** Maximum power and ventilation as well as ventilation at 20% and 50% of maximum ventilation.

	Young (n = 40)		Elderly (n = 40)	
	Women (n = 20)	Men (n = 20)	Women (n = 20)	Men (n = 20)
Max. power (W)	169 ± 35 (100–250)	269 ± 49 (175–350)	106 ± 23 (75–150)	179 ± 27 (125–225)
Max. ventilation (L/min)	83.6 ± 16.3 (54.7–121.7)	126.2 ± 22.3 (88.3–163.9)	51.9 ± 12.0 (27.9–76.1)	88.5 ± 20.8 (46.7–126.7)
20% maximum ventilation (L/min)	16.7 ± 3.2 (10.9–24.3)	25.2 ± 4.5 (17.7–32.8)	10.4 ± 2.4 (5.6–15.2)	17.7 ± 4.2 (9.3–25.3)
50% maximum ventilation (L/min)	41.8 ± 8.2 (27.3–60.9)	63.1 ± 11.1 (44.1–82.0)	26.0 ± 6.0 (13.9–38.0)	44.2 ± 10.4 (23.3–63.3)

All data are mean values ± standard deviation as well as range (in parentheses). Differences between the age groups as well as between men and women are always significant (p < 0.001, each).

### Aerosol particle emission

**Figure 2:**
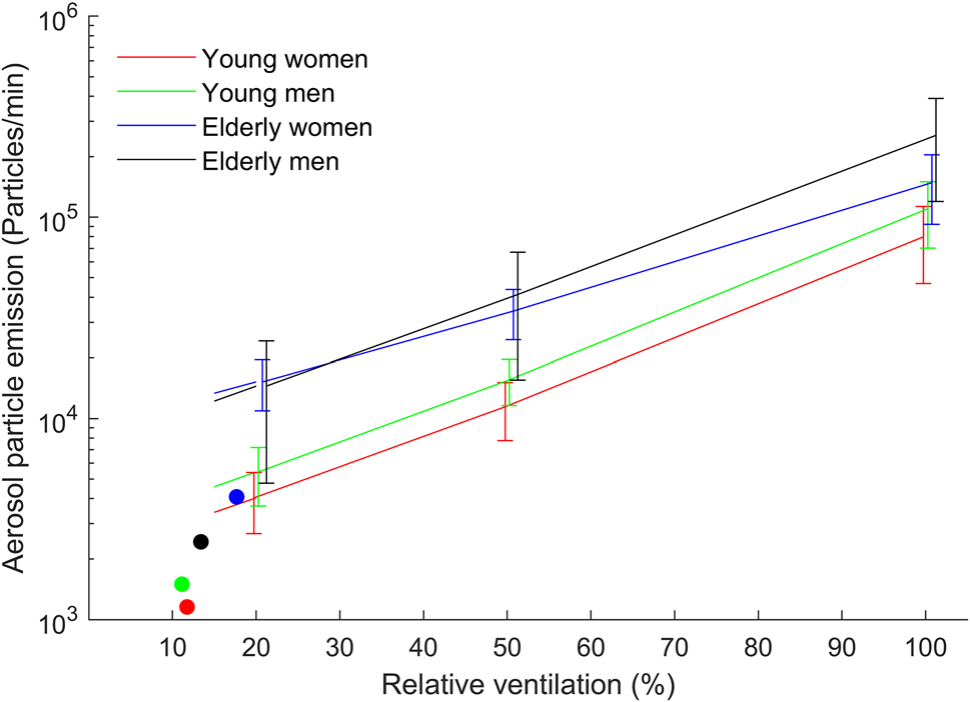
Aerosol particle emission (Particles/min) in dependence of the relative ventilation for the four groups. Legend: Resting values are shown with round markers. Relative ventilation was defined as fraction of ventilation relative to the maximum ventilation reached at maximum exercise intensity.

As reported previously^4^, aerosol particle emission differed between young and older subjects at rest and during exercise. It is plotted as function of relative ventilation in Fig. 2, instead of relative workload as previously^4^. In the present analysis we focus on ventilation, since primarily ventilation can be assumed to be associated with lung function in healthy subjects.

### Association between aerosol particle emission and lung function

To identify predictors of aerosol particle emission, we compared aerosol particle mission at 20%, 40% and 50% of maximum ventilation with sex, body mass index (BMI), smoking history, MEF25, MEF50, FVC, and impulse oscillometry parameters. Due to their collinearity, the associations with impulse oscillometry parameters were tested in separate analyses. Sex, BMI, smoking history, MEF50 and FVC were never linked to particle emission, in contrast to MEF25 (p < 0.05). Among quiet breathing impulse oscillometry parameters, only reactance at 5 Hz (X5) was associated with emission (p < 0.05), among vital capacity manoeuvre impulse oscillometry parameters only expiratory resistance at 5 Hz (R5ex15) (p < 0.05); their associations are illustrated in Table S1.

After having identified R5ex15 and X5 as single impulse oscillometry parameters related to emission, they were combined in a further regression analysis, again keeping sex, BMI, smoking history, MEF25, MEF50 and FVC as covariates. For percentages of maximum ventilation between 20 and 50%, both parameters were significantly associated (p < 0.05) with aerosol particle emission as shown in Table 4 (left part). To reveal whether impulse oscillometry parameters were related to emission independently of age, we repeated these analyses with age group as additional predictor (Table 4 right). The relationship of particle emission to impulse oscillometry parameters was maintained, while at the same time an additional dependence from age group appeared (p = 0.029, see Table S2). When repeating the analysis without the two impulse oscillometry parameters, the standardised regression coefficient of age group increased from 0.419 to 0.582 (p = 0.002).

**Table 4:** Standardised regression coefficients.

		Without age group			With age group		
		20% maximum ventilation	40% maximum ventilation	50% maximum ventilation	20% maximum ventilation	40% maximum ventilation	50% maximum ventilation
Age group	beta	–	–	–	0.419	0.395	0.376
	p-value	–	–	–	0.029	0.039	0.05
	95% confidence interval for beta	–	–	–	0.045–0.793	0.021–0.769	− 0.001 to 0.753
R5ex15 (kPa/L/s)	beta	0.267	0.275	0.275	0.252	0.260	0.261
	p-value	0.011	0.009	0.009	0.014	0.011	0.011
	95% confidence interval for beta	0.063–0.471	0.071–0.480	0.071–0.480	0.053–0.452	0.061–0.458	0.061 to 0.461
X5 (kPa/L/s)	beta	0.357	0.379	0.386	0.249	0.277	0.289
	p-value	0.002	0.001	0.001	0.042	0.024	0.020
	95% confidence interval for beta	0.132–0.583	0.154–0.604	0.160–0.612	0.009–0.489	0.037–0.517	0.047 to 0.531

Standardised regression coefficients beta, p-value and their confidence intervals from linear regression analyses of aerosol particle emission at 20%, 40% and 50% of maximum ventilation as dependent variables. Analysis of R5ex15 and X5 was done with and without including age group. Sex, BMI, smoking history, MEF25, MEF50 and FVC were always kept as covariates (coefficients not shown).

**Figure 3:**
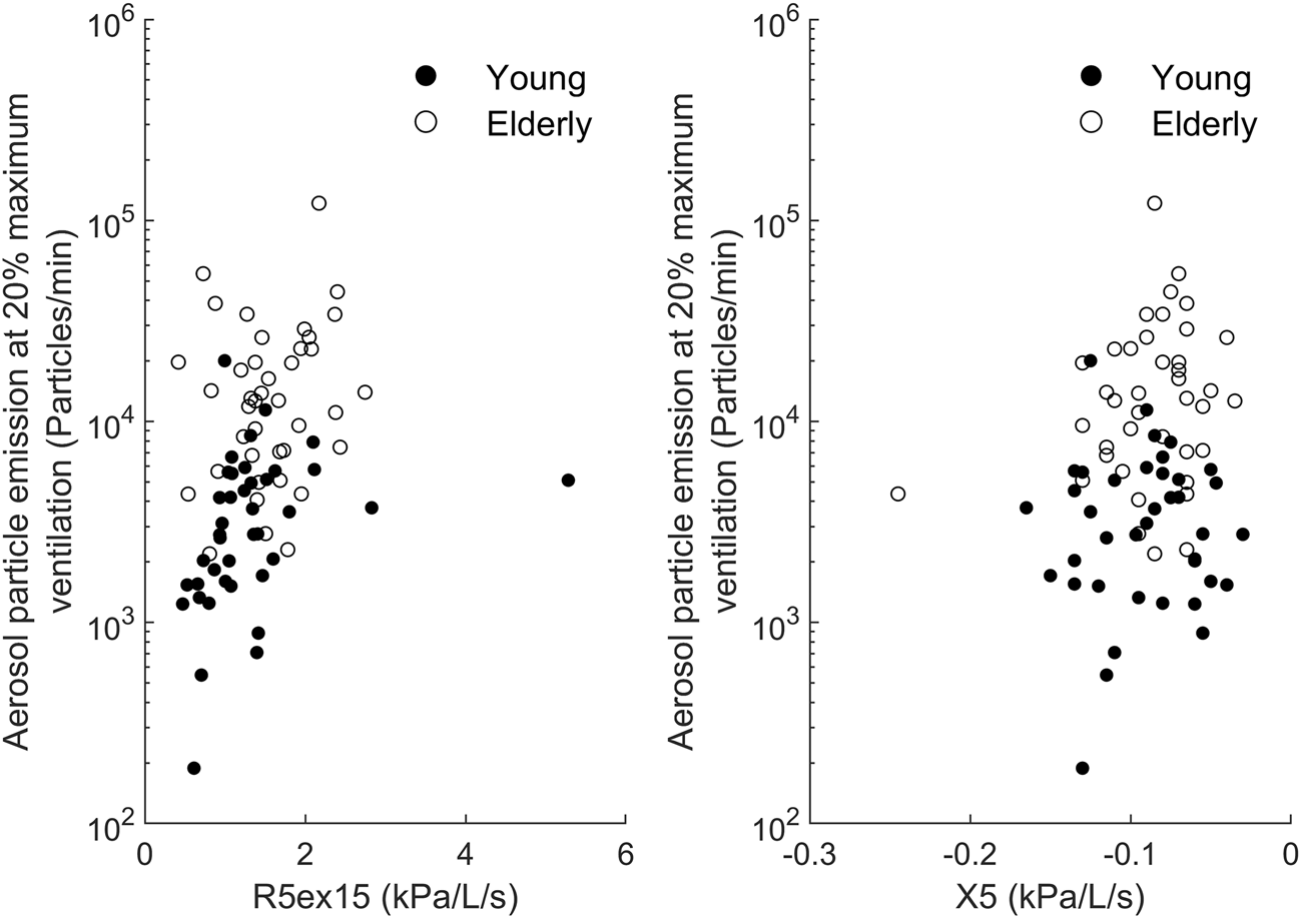
Relationship between (**A**) the low-frequency resistance of the last 15% of vital capacity (R5ex15) and (**B**) X5 and aerosol particle emission at relative maximum ventilations of 20%. There were significant correlations over the total group of subjects as well as within the younger subjects for R5ex15 but not the elderly.

Figure 3A,B show the associations between R5ex15 and X5, respectively, and emission at 20% of maximum ventilation, suggesting that the association with impulse oscillometry parameters was stronger in the younger compared to the older group. This was confirmed by a separate analysis of groups, revealing a significant (p = 0.009) association with R5ex15 in the younger but not the older subjects. X5 was not significantly related in the two separate groups, although there was a tendency in the elderly (p = 0.076).

### Sensitivity analysis

When omitting the subject with the extreme value of R5ex15 (Fig. 3A), its association with emission was still significant (p = 0.017). Omission of the extreme value of X5 (see Fig. 3B) also did not lead to a different result. When excluding those of the 80 lung-healthy subjects in whom the ratio FEV_1_/FVC was below LLN, the association of emission at 20% of maximum ventilation with R5ex15 (p = 0.020) and X5 (p = 0.019) as well as age group (p = 0.004) remained statistically significant.

## Discussion

In the present study we found a consistent and robust association between aerosol particle emission at different ventilation rates during exercise and two parameters of airway function (reactance (X5) and expiratory resistance (R5ex15) at 5 Hz) in lung-healthy participants aged 20 to 76 years. In addition to reactance at 5 Hz assessed via impulse oscillometry, we identified a resistance parameter at 5 Hz at a defined level of lung volume. The association with this parameter was even found within the group of the younger subjects. Thus, our study provides specific lung function parameters associated with endogenous aerosol particle generation of the lung that do not simply reflect the difference in age. These lung function parameters could partially explain the previously reported about twofold higher aerosol particle emission in the older compared to the younger subjects both at rest and during exercise^4^.

Exhaled aerosol particles are generated in the small airways via cyclic collapsing and reopening^1,6^, suggesting that small airway function affects particle generation. Maximum expiratory flow rates at lower lung volumes are considered as indicative of small airway function but did not show robust associations with particle emission. The presence of exhaled aerosol particles requires both the collapse-reopening generating process and adequate ventilation transporting the particles into the expired air. Maximum expiratory flow rates measured during forced manoeuvres may only be poorly associated with the conditions of collapse-reopening during normal ventilation. These characteristics might be better described by lung function parameters obtained during moderate flow rates or even resting ventilation, as provided by impulse oscillometry. Correspondingly we performed impulse oscillometry not only during resting ventilation, but also a deep expiration at moderate flow rate.

In accordance with established interpretations^10^ we identified the low-frequency (5 Hz) resistance at low lung volume during the expiratory manoeuvre as robust predictor of particle production during moderate ventilation rates. In addition, we found the reactance at 5 Hz to be relevant, in accordance with the interpretation that this is an indicator of airway elasticity^10^ which should affect aerosol production in the collapse/re-opening cycle. Often, the difference between resistance values at 5 and 20 Hz is considered as describing peripheral airway characteristics but we did not find this parameter to be relevant, possibly due to the fact that the range of variation between subjects was relatively small, as we included only lung-healthy subjects. The significant difference between the two age groups regarding this parameter was in line with the expectations^15^ and underlined the validity of our measurements. In all these interpretations, however, it should be taken into account that most of the evidence for the relationship between IOS parameters and peripheral airway function is indirect and currently still hypothetical^16^.

We used a vital capacity manoeuvre addressing the variation of resistance with lung volume at non-maximum flow rates. This was quantified as average resistance over the final part of a deep expiration, using different percentages of vital capacity. We reasoned that the low volumes would be most informative but also prone to artefacts, whereas a broader range of volumes would lead to more robust average values but bare the risk of integrating over non-informative resistance values at high lung volumes. The Table S1 showed that the correlation between vital capacity manoeuvre resistance parameters and particle emission at different maximum ventilation rates was maximal at about 15% of individual vital capacity, thereby underlining the assumption that functional parameters at relative low lung volumes are needed.

The present analysis extends a study that addressed the difference in aerosol particle emissions between younger and older subjects during rest and exercise^4^, giving a detailed characterization of aerosol particle generation in dependence from workload during exercise. Workload is suitable to address everyday conditions as it is more easily measured than ventilation, although both are related as illustrated in Fig. S1. In the present analysis we used ventilation rate as more adequate regarding aerosol particle production. To facilitate the comparison of individual data, ventilation rates were expressed as percentages of individual maximum ventilation.

As shown in Table 3, ventilation rates of 20% corresponded to light exercise, depending on sex and age, whereas 50% corresponded to heavy exercise under everyday circumstances. The correlation with impulse oscillometry vital capacity manoeuvre parameters was even seen at 15% of maximum exercise which corresponded to values close to resting ventilation (data not shown). The correlations with impulse oscillometry parameters were consistently present up to 50% of maximum ventilation.

The association between impulse oscillometry vital capacity manoeuvre parameters and particle emission was particularly apparent within the younger subjects, in contrast to the older subjects. Particle emission in the elderly was higher than predicted by the extrapolation from the younger subjects (Fig. 3). This strongly suggests that there are further determinants of aerosol particle generation from the lung. Their assessment might require more direct indicators of peripheral airway characteristics, including their variation over the breathing cycle. It might be that the surface properties of peripheral airways change with age, although the presently available data^17^ only suggest a link with a presence of airway diseases. It remains a topic for further research to identify the full spectrum of alterations underlying the consistently higher aerosol particle emission from the lung in older compared to younger subjects.

### Limitations

We assessed a variety of lung function indices with the aim to identify the most adequate ones related to the increase of aerosol particle emission with age. There may be other, more direct and until now unrecognized indices of peripheral airway function that are causally related to aerosol particle generation. Their determination might include high resolution or optical computer tomograms at different levels of lung inflation as performed, for example, for bronchial provocation testing^18^. It might also be of interest to include subjects with airway diseases in order to cover a broader range of function and dysfunction.

## Conclusion

In lung-healthy subjects, the rate of particle emission from the lung assessed during light to moderate exercise was associated with indices of airway function from impulse oscillometry that were assessed at low lung volumes and during resting ventilation. A higher value of low-frequency resistance (5 Hz) was linked to higher particle emission, probably indicating increased collapse-reopening dynamics of peripheral airways responsible for endogenous aerosol particle generation. Thus, individual differences in aerosol particle emission, either within young subjects or between older and younger subjects seemed to be related to measurable differences in peripheral airway characteristics, although the dependence on age seems to require further mechanisms.

### Supplementary Information

Supplementary Information 1.

Supplementary Information 2.

## Data Availability

All data is included in the Supplementary Information.
