# Supplementary material for: Indices of airway resistance and reactance from impulse oscillometry correlate with aerosol particle emission in different age groups
Source: Sci Rep. 2024 Feb 26;14:4644. doi: 10.1038/s41598-024-55117-2 (PMC10897442; doi:10.1038/s41598-024-55117-2)
Supplement: Supplementary file 1 — Supplementary Information 1. [file 41598_2024_55117_MOESM1_ESM.pdf]

Supplementary Information

Indices of airway resistance and reactance from impulse oscillometry correlate with aerosol particle emission in different age groups

Benedikt Schumm, Stephanie Bremer, Katharina Knödlseder, Martin Schönfelder, Rainer Hain, Luisa Semmler, Elke Lorenz, Henning Wackerhage, Christian J. Kähler and Rudolf Jörres

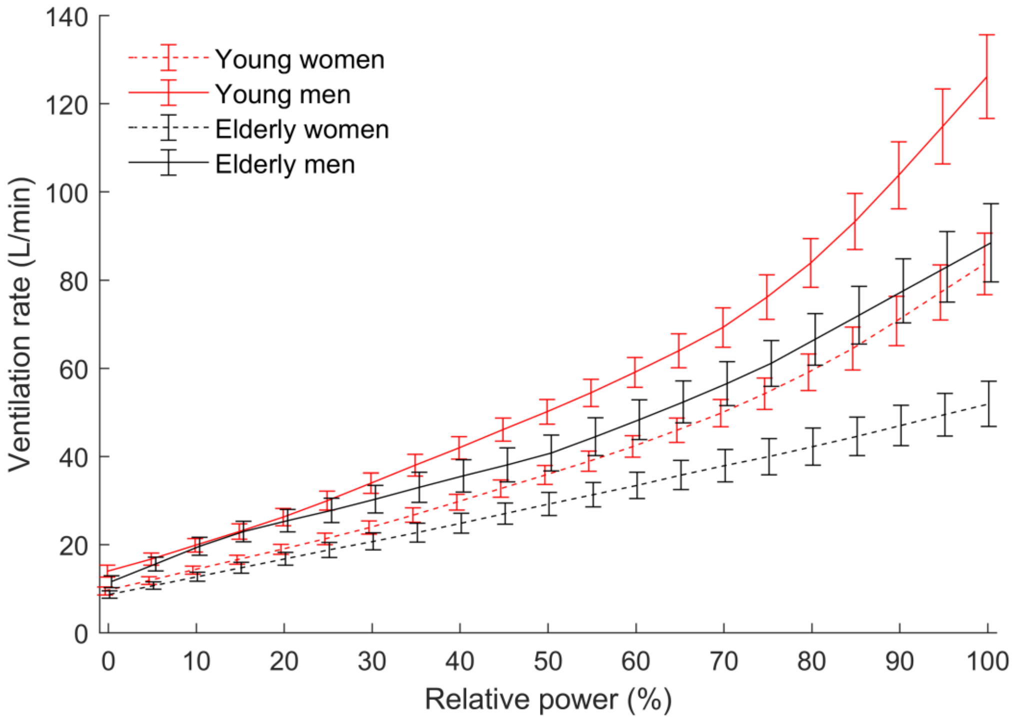

Figure S1: Ventilation rate versus relative power for the 4 groups with 95% confidence interval

Table S1: Heatmap showing the p-values for the different regression analyses illustrating the association between aerosol particle emission at different relative ventilation rates and impulse oscillometry vital capacity maneuver values in terms of resistances at 5 Hz at different % of vital capacity and the reactance value at 5 Hz. Light green indicates a high degree of significance, green a tendency toward significance and rose values to no significance.

Relative ventilation rate in % of maximum ventilation	15	20	25	30	40	50
**R5ex50**	0.127	0.119	0.112	0.106	0.096	0.089
**R5ex40**	0.101	0.095	0.09	0.086	0.08	0.076
**R5ex25**	0.073	0.069	0.067	0.065	0.063	0.063
**R5ex15**	0.045	0.043	0.042	0.041	0.04	0.042
**R5ex5**	0.074	0.074	0.074	0.075	0.079	0.087
**X5**	0.011	0.01	0.009	0.008	0.006	0.005

Table S2: Exemplary linear regression analysis at 20% of maximum ventilation

	Regression coefficient B	Standard error	Standardised coefficient Beta	95% confidence interval		p-value
				Lower limit	Upper limit	
Age group	0.825	0.369	0.419	0.089	1.561	0.029
Sex	-0.088	0.399	-0.045	-0.883	0.708	0.826
BMI (kg/m²)	0.058	0.033	0.198	-0.007	0.124	0.080
Smoking history	0.032	0.21	0.016	-0.387	0.451	0.879
FVC (L)	-0.04	0.194	-0.05	-0.427	0.347	0.838
MEF25 (L/s)	-0.057	0.2	-0.059	-0.456	0.342	0.777
MEF50 (L/s)	0.069	0.103	0.12	-0.137	0.275	0.508
R5ex15 (kPa/L/s)	0.36	0.142	0.252	0.076	0.645	0.014
X5 (kPa/L/s)	7.151	3.452	0.249	0.262	14.039	0.042
