# Supplementary material for: Indices of airway resistance and reactance from impulse oscillometry correlate with aerosol particle emission in different age groups
Source: Sci Rep. 2024 Feb 26;14:4644. doi: 10.1038/s41598-024-55117-2 (PMC10897442; doi:10.1038/s41598-024-55117-2)
Supplement: Supplementary file 2 — Supplementary Information 2. [file 41598_2024_55117_MOESM2_ESM.pdf]

| Rest_APE_rVE | Rest_VE | rel_resting_VE | Inter_APE_rVE | Slope_APE_rVE | APE_rVE_15 | APE_rVE_20 | APE_rVE_25 | APE_rVE_30 | APE_rVE_40 | APE_rVE_50 | APE_rVE_75 | APE_rVE_100 | FEV1_include | FVC_include |
|---|---|---|---|---|---|---|---|---|---|---|---|---|---|---|
| 5.91 | 11.76 | 0.11 | 6.92 | 3.87 | 7.50 | 7.70 | 7.89 | 8.08 | 8.47 | 8.86 | 9.82 | 10.79 | 1 | 1 |
| 6.71 | 11.68 | 0.20 | 6.93 | 3.79 | 7.50 | 7.69 | 7.88 | 8.07 | 8.45 | 8.83 | 9.78 | 10.72 | 0 | 1 |
| 6.99 | 8.64 | 0.10 | 7.65 | 3.49 | 8.17 | 8.34 | 8.52 | 8.69 | 9.04 | 9.39 | 10.26 | 11.13 | 1 | 1 |
| 7.00 | 8.87 | 0.14 | 7.28 | 2.99 | 7.73 | 7.88 | 8.03 | 8.18 | 8.48 | 8.78 | 9.53 | 10.28 | 0 | 1 |
| 5.97 | 7.15 | 0.08 | 7.29 | 3.57 | 7.83 | 8.00 | 8.18 | 8.36 | 8.72 | 9.07 | 9.97 | 10.86 | 1 | 1 |
| 8.06 | 6.82 | 0.07 | 8.11 | 3.64 | 8.65 | 8.83 | 9.02 | 9.20 | 9.56 | 9.93 | 10.84 | 11.75 | 1 | 1 |
| 6.53 | 12.54 | 0.14 | 6.66 | 4.05 | 7.27 | 7.47 | 7.68 | 7.88 | 8.28 | 8.69 | 9.70 | 10.71 | 1 | 1 |
| 8.09 | 9.46 | 0.17 | 7.74 | 3.20 | 8.22 | 8.38 | 8.54 | 8.70 | 9.02 | 9.34 | 10.14 | 10.94 | 1 | 1 |
| 5.70 | 12.73 | 0.15 | 4.39 | 6.53 | 5.37 | 5.70 | 6.02 | 6.35 | 7.00 | 7.66 | 9.29 | 10.92 | 1 | 1 |
| 7.79 | 7.89 | 0.12 | 8.51 | 4.08 | 9.12 | 9.32 | 9.52 | 9.73 | 10.14 | 10.54 | 11.56 | 12.58 | 1 | 1 |
| 7.46 | 5.55 | 0.10 | 7.74 | 2.80 | 8.16 | 8.30 | 8.44 | 8.58 | 8.86 | 9.14 | 9.83 | 10.53 | 1 | 1 |
| 7.65 | 8.36 | 0.10 | 8.30 | 2.58 | 8.69 | 8.82 | 8.95 | 9.08 | 9.33 | 9.59 | 10.24 | 10.88 | 1 | 1 |
| 8.12 | 7.29 | 0.14 | 9.29 | 1.36 | 9.49 | 9.56 | 9.63 | 9.69 | 9.83 | 9.96 | 10.30 | 10.64 | 1 | 1 |
| 8.57 | 14.62 | 0.12 | 9.31 | 2.85 | 9.74 | 9.88 | 10.02 | 10.17 | 10.45 | 10.74 | 11.45 | 12.16 | 1 | 1 |
| 8.57 | 11.23 | 0.15 | 7.63 | 3.22 | 8.11 | 8.27 | 8.43 | 8.59 | 8.91 | 9.23 | 10.04 | 10.84 | 1 | 1 |
| 6.34 | 8.01 | 0.12 | 6.83 | 2.97 | 7.28 | 7.43 | 7.58 | 7.73 | 8.02 | 8.32 | 9.06 | 9.81 | 1 | 1 |
| 8.35 | 13.21 | 0.13 | 7.54 | 3.89 | 8.12 | 8.32 | 8.51 | 8.71 | 9.10 | 9.49 | 10.46 | 11.43 | 1 | 1 |
| 7.96 | 10.71 | 0.14 | 8.04 | 4.01 | 8.64 | 8.84 | 9.04 | 9.24 | 9.64 | 10.04 | 11.05 | 12.05 | 1 | 1 |
| 6.44 | 11.43 | 0.12 | 5.52 | 5.11 | 6.29 | 6.54 | 6.80 | 7.05 | 7.57 | 8.08 | 9.35 | 10.63 | 1 | 1 |
| 8.09 | 15.44 | 0.15 | 8.73 | 3.06 | 9.19 | 9.34 | 9.49 | 9.65 | 9.95 | 10.26 | 11.02 | 11.79 | 1 | 1 |
| 8.38 | 10.35 | 0.11 | 8.33 | 4.32 | 8.98 | 9.20 | 9.41 | 9.63 | 10.06 | 10.49 | 11.57 | 12.65 | 1 | 1 |
| 8.27 | 9.36 | 0.16 | 9.75 | 1.66 | 10.00 | 10.09 | 10.17 | 10.25 | 10.42 | 10.58 | 11.00 | 11.41 | 1 | 1 |
| 7.52 | 13.06 | 0.16 | 8.67 | 3.18 | 9.14 | 9.30 | 9.46 | 9.62 | 9.94 | 10.26 | 11.05 | 11.85 | 1 | 1 |
| 6.41 | 7.18 | 0.09 | 5.92 | 4.76 | 6.64 | 6.87 | 7.11 | 7.35 | 7.83 | 8.30 | 9.49 | 10.68 | 1 | 1 |
| 7.63 | 8.34 | 0.09 | 8.14 | 3.00 | 8.59 | 8.74 | 8.89 | 9.04 | 9.33 | 9.63 | 10.38 | 11.13 | 1 | 1 |
| 7.22 | 10.94 | 0.15 | 7.58 | 3.59 | 8.12 | 8.30 | 8.48 | 8.66 | 9.02 | 9.38 | 10.28 | 11.17 | 1 | 1 |
| 10.02 | 9.94 | 0.36 | 8.70 | 2.30 | 9.05 | 9.16 | 9.28 | 9.39 | 9.62 | 9.85 | 10.43 | 11.01 | 1 | 1 |
| 8.96 | 7.90 | 0.18 | 9.03 | 2.68 | 9.43 | 9.57 | 9.70 | 9.84 | 10.10 | 10.37 | 11.04 | 11.71 | 1 | 1 |
| 6.70 | 10.37 | 0.08 | 7.41 | 3.31 | 7.91 | 8.07 | 8.24 | 8.41 | 8.74 | 9.07 | 9.90 | 10.73 | 1 | 1 |
| 7.06 | 14.80 | 0.12 | 6.63 | 4.94 | 7.37 | 7.62 | 7.87 | 8.12 | 8.61 | 9.10 | 10.34 | 11.57 | 1 | 1 |
| 7.74 | 13.97 | 0.19 | 6.91 | 4.04 | 7.51 | 7.72 | 7.92 | 8.12 | 8.52 | 8.93 | 9.94 | 10.95 | 1 | 1 |
| 6.69 | 11.44 | 0.10 | 7.66 | 4.18 | 8.28 | 8.49 | 8.70 | 8.91 | 9.33 | 9.75 | 10.79 | 11.83 | 1 | 1 |
| 8.68 | 9.47 | 0.16 | 8.66 | 2.57 | 9.05 | 9.17 | 9.30 | 9.43 | 9.69 | 9.94 | 10.59 | 11.23 | 1 | 1 |
| 7.12 | 5.83 | 0.12 | 8.26 | 3.59 | 8.80 | 8.98 | 9.16 | 9.33 | 9.69 | 10.05 | 10.95 | 11.85 | 1 | 1 |
| 6.60 | 10.38 | 0.16 | 7.33 | 2.85 | 7.76 | 7.90 | 8.05 | 8.19 | 8.47 | 8.76 | 9.47 | 10.18 | 1 | 1 |
| 8.67 | 11.23 | 0.14 | 9.00 | 2.02 | 9.31 | 9.41 | 9.51 | 9.61 | 9.81 | 10.01 | 10.52 | 11.03 | 1 | 1 |
| 8.93 | 10.23 | 0.17 | 9.33 | 3.05 | 9.79 | 9.94 | 10.10 | 10.25 | 10.55 | 10.86 | 11.62 | 12.38 | 1 | 1 |
| 8.61 | 10.81 | 0.18 | 9.08 | 3.29 | 9.57 | 9.73 | 9.90 | 10.06 | 10.39 | 10.72 | 11.54 | 12.36 | 0 | 1 |
| 7.96 | 17.34 | 0.13 | 8.43 | 2.48 | 8.80 | 8.92 | 9.05 | 9.17 | 9.42 | 9.67 | 10.29 | 10.91 | 1 | 1 |
| 8.81 | 12.56 | 0.10 | 8.52 | 2.89 | 8.95 | 9.09 | 9.24 | 9.38 | 9.67 | 9.96 | 10.68 | 11.40 | 1 | 1 |
| 6.87 | 13.62 | 0.11 | 7.91 | 2.81 | 8.33 | 8.47 | 8.62 | 8.76 | 9.04 | 9.32 | 10.02 | 10.73 | 1 | 1 |
| 6.97 | 9.02 | 0.27 | 6.72 | 2.03 | 7.02 | 7.12 | 7.22 | 7.33 | 7.53 | 7.73 | 8.24 | 8.75 | 1 | 1 |
| 8.91 | 10.51 | 0.09 | 10.94 | 3.26 | 11.43 | 11.59 | 11.75 | 11.91 | 12.24 | 12.57 | 13.38 | 14.20 | 1 | 1 |
| 7.60 | 15.01 | 0.14 | 8.03 | 3.71 | 8.58 | 8.77 | 8.95 | 9.14 | 9.51 | 9.88 | 10.81 | 11.73 | 1 | 1 |
| 8.51 | 11.97 | 0.16 | 9.02 | 4.20 | 9.65 | 9.86 | 10.07 | 10.29 | 10.71 | 11.13 | 12.18 | 13.23 | 1 | 1 |
| 6.96 | 7.09 | 0.07 | 8.31 | 3.41 | 8.82 | 8.99 | 9.16 | 9.33 | 9.67 | 10.01 | 10.87 | 11.72 | 1 | 1 |
| 8.77 | 7.88 | 0.13 | 9.76 | 2.35 | 10.11 | 10.23 | 10.34 | 10.46 | 10.70 | 10.93 | 11.52 | 12.11 | 1 | 1 |
| 7.93 | 22.11 | 0.16 | 8.50 | 2.71 | 8.91 | 9.04 | 9.18 | 9.31 | 9.59 | 9.86 | 10.54 | 11.21 | 1 | 1 |
| 6.42 | 5.69 | 0.07 | 7.13 | 3.30 | 7.63 | 7.79 | 7.96 | 8.12 | 8.45 | 8.78 | 9.61 | 10.43 | 1 | 1 |
| 7.40 | 11.58 | 0.23 | 9.77 | 2.12 | 10.09 | 10.19 | 10.30 | 10.41 | 10.62 | 10.83 | 11.36 | 11.89 | 1 | 1 |
| 9.22 | 14.62 | 0.10 | 8.64 | 2.64 | 9.04 | 9.17 | 9.30 | 9.44 | 9.70 | 9.97 | 10.63 | 11.29 | 1 | 1 |
| 6.52 | 8.81 | 0.09 | 7.60 | 4.10 | 8.22 | 8.42 | 8.63 | 8.83 | 9.24 | 9.66 | 10.68 | 11.71 | 1 | 1 |
| 7.72 | 11.55 | 0.13 | 7.91 | 3.17 | 8.38 | 8.54 | 8.70 | 8.86 | 9.17 | 9.49 | 10.28 | 11.07 | 1 | 1 |
| 6.77 | 15.90 | 0.11 | 7.55 | 4.66 | 8.25 | 8.48 | 8.71 | 8.95 | 9.41 | 9.88 | 11.04 | 12.21 | 1 | 1 |
| 8.49 | 7.36 | 0.16 | 9.19 | 1.91 | 9.48 | 9.57 | 9.67 | 9.77 | 9.96 | 10.15 | 10.62 | 11.10 | 1 | 1 |
| 9.04 | 9.11 | 0.25 | 8.99 | 2.21 | 9.32 | 9.43 | 9.54 | 9.65 | 9.88 | 10.10 | 10.65 | 11.20 | 1 | 1 |
| 7.80 | 11.12 | 0.10 | 8.76 | 3.37 | 9.26 | 9.43 | 9.60 | 9.77 | 10.11 | 10.44 | 11.28 | 12.13 | 1 | 1 |
| 7.89 | 5.16 | 0.09 | 10.16 | 2.57 | 10.55 | 10.68 | 10.81 | 10.93 | 11.19 | 11.45 | 12.09 | 12.73 | 1 | 1 |
| 7.88 | 11.28 | 0.11 | 8.71 | 3.97 | 9.31 | 9.51 | 9.71 | 9.91 | 10.30 | 10.70 | 11.69 | 12.69 | 1 | 1 |
| 8.14 | 6.96 | 0.17 | 7.78 | 2.96 | 8.22 | 8.37 | 8.52 | 8.66 | 8.96 | 9.25 | 9.99 | 10.73 | 1 | 1 |
| 9.37 | 9.27 | 0.20 | 9.37 | 2.51 | 9.74 | 9.87 | 9.99 | 10.12 | 10.37 | 10.62 | 11.25 | 11.88 | 1 | 1 |
| 7.34 | 10.38 | 0.14 | 6.78 | 3.48 | 7.30 | 7.47 | 7.65 | 7.82 | 8.17 | 8.52 | 9.38 | 10.25 | 1 | 1 |
| 8.68 | 14.68 | 0.18 | 9.36 | 3.37 | 9.87 | 10.04 | 10.21 | 10.37 | 10.71 | 11.05 | 11.89 | 12.74 | 1 | 1 |
| 7.76 | 11.10 | 0.19 | 7.40 | 3.88 | 7.98 | 8.17 | 8.37 | 8.56 | 8.95 | 9.34 | 10.31 | 11.28 | 1 | 1 |
| 7.47 | 8.28 | 0.11 | 8.12 | 4.31 | 8.77 | 8.99 | 9.20 | 9.42 | 9.85 | 10.28 | 11.36 | 12.44 | 1 | 1 |
| 6.94 | 9.41 | 0.10 | 6.38 | 4.00 | 6.98 | 7.18 | 7.38 | 7.58 | 7.98 | 8.38 | 9.38 | 10.37 | 1 | 1 |
| 7.23 | 14.63 | 0.11 | 6.79 | 5.04 | 7.54 | 7.80 | 8.05 | 8.30 | 8.81 | 9.31 | 10.57 | 11.83 | 1 | 1 |
| 7.03 | 13.07 | 0.10 | 7.32 | 4.24 | 7.96 | 8.17 | 8.38 | 8.59 | 9.02 | 9.44 | 10.50 | 11.56 | 1 | 1 |
| 6.29 | 13.81 | 0.09 | 7.48 | 3.58 | 8.01 | 8.19 | 8.37 | 8.55 | 8.91 | 9.26 | 10.16 | 11.05 | 1 | 1 |
| 7.69 | 18.66 | 0.15 | 8.07 | 5.14 | 8.84 | 9.10 | 9.36 | 9.61 | 10.13 | 10.64 | 11.93 | 13.21 | 1 | 1 |
| 7.21 | 10.18 | 0.14 | 7.66 | 4.55 | 8.34 | 8.57 | 8.80 | 9.02 | 9.48 | 9.93 | 11.07 | 12.21 | 1 | 1 |
| 5.60 | 8.81 | 0.14 | 5.97 | 6.42 | 6.94 | 7.26 | 7.58 | 7.90 | 8.54 | 9.18 | 10.79 | 12.39 | 1 | 1 |
| 8.29 | 13.77 | 0.14 | 9.08 | 1.94 | 9.37 | 9.46 | 9.56 | 9.66 | 9.85 | 10.04 | 10.53 | 11.01 | 1 | 1 |
| 6.84 | 17.04 | 0.11 | 6.77 | 4.38 | 7.43 | 7.65 | 7.86 | 8.08 | 8.52 | 8.96 | 10.05 | 11.15 | 1 | 1 |
| 7.15 | 7.40 | 0.06 | 8.42 | 4.29 | 9.07 | 9.28 | 9.49 | 9.71 | 10.14 | 10.57 | 11.64 | 12.71 | 1 | 1 |
| 7.45 | 16.89 | 0.13 | 7.60 | 4.46 | 8.26 | 8.49 | 8.71 | 8.93 | 9.38 | 9.83 | 10.94 | 12.05 | 1 | 1 |
| 9.39 | 14.17 | 0.17 | 9.39 | 3.57 | 9.92 | 10.10 | 10.28 | 10.46 | 10.81 | 11.17 | 12.06 | 12.96 | 1 | 1 |
| 6.34 | 10.31 | 0.10 | 7.05 | 3.40 | 7.56 | 7.73 | 7.90 | 8.07 | 8.41 | 8.75 | 9.60 | 10.45 | 1 | 1 |
| 8.02 | 4.01 | 0.09 | 8.87 | 2.50 | 9.24 | 9.37 | 9.50 | 9.62 | 9.87 | 10.12 | 10.75 | 11.37 | 1 | 1 |
| 7.40 | 13.09 | 0.08 | 7.41 | 5.59 | 8.25 | 8.53 | 8.81 | 9.09 | 9.65 | 10.21 | 11.61 | 13.01 | 1 | 1 |

| FEV1_FVC_include | FEV1FVC_include | FEV1_FVC_FEV1FVC_include | Agegroup_1young_2old | BMIgroup_1n2o | Group | Age | Sex_1W_2M | Height | Weight | BMI | FEV1FEV1 | MEF25FVC | MEF50FVC | MEF25MEF25 | MEF50MEF50 | FEV1 | FVC | MEF25 |
|---|---|---|---|---|---|---|---|---|---|---|---|---|---|---|---|---|---|---|
| 1 | 1 | 1 | 1 | 2 | 4 | 33 | 2 | 185.0 | 85.6 | 25.011 | 1.035 | 0.376 | 0.933 | 1.179 | 1.012 | 4.990 | 6.120 | 2.300 |
| 0 | 1 | 1 | 2 | 2 | 2 | 69 | 2 | 180.0 | 82.4 | 25.432 | 0.569 | 0.058 | 0.248 | 0.286 | 0.199 | 1.900 | 3.470 | 0.200 |
| 1 | 1 | 1 | 1 | 2 | 3 | 28 | 1 | 170.2 | 78.4 | 27.064 | 1.011 | 0.556 | 1.041 | 1.294 | 0.942 | 3.610 | 4.190 | 2.330 |
| 0 | 1 | 0 | 1 | 1 | 3 | 24 | 1 | 151.3 | 51.9 | 22.672 | 0.678 | 0.209 | 0.500 | 0.433 | 0.384 | 1.920 | 3.260 | 0.680 |
| 1 | 1 | 1 | 1 | 1 | 4 | 30 | 2 | 180.0 | 77.3 | 23.858 | 1.030 | 0.633 | 1.226 | 1.699 | 1.164 | 4.750 | 5.260 | 3.330 |
| 1 | 1 | 1 | 1 | 1 | 3 | 30 | 1 | 167.5 | 61.6 | 21.956 | 0.892 | 0.408 | 1.405 | 0.880 | 1.115 | 3.050 | 3.580 | 1.460 |
| 1 | 1 | 1 | 1 | 2 | 3 | 22 | 1 | 168.3 | 73.9 | 26.090 | 1.174 | 0.476 | 0.998 | 1.107 | 1.021 | 4.190 | 4.770 | 2.270 |
| 1 | 1 | 1 | 1 | 1 | 3 | 24 | 1 | 162.3 | 58.7 | 22.284 | 0.933 | 0.499 | 0.949 | 1.017 | 0.776 | 3.060 | 3.690 | 1.840 |
| 1 | 1 | 1 | 1 | 1 | 3 | 22 | 1 | 164.0 | 62.4 | 23.200 | 1.124 | 0.705 | 1.354 | 1.469 | 1.202 | 3.800 | 4.040 | 2.850 |
| 1 | 1 | 1 | 2 | 1 | 1 | 60 | 1 | 163.4 | 61.3 | 22.959 | 1.107 | 0.124 | 0.821 | 0.727 | 0.866 | 2.790 | 3.860 | 0.480 |
| 1 | 1 | 1 | 2 | 2 | 1 | 70 | 1 | 162.8 | 68.8 | 25.959 | 1.248 | 0.080 | 0.655 | 0.717 | 0.794 | 2.770 | 4.120 | 0.330 |
| 1 | 1 | 1 | 1 | 2 | 3 | 22 | 1 | 156.8 | 72.0 | 29.285 | 0.984 | 0.835 | 1.607 | 1.539 | 1.203 | 3.020 | 3.280 | 2.740 |
| 1 | 1 | 1 | 2 | 2 | 1 | 64 | 1 | 165.5 | 70.9 | 25.885 | 1.040 | 0.182 | 0.792 | 1.103 | 0.770 | 2.580 | 3.510 | 0.640 |
| 1 | 0 | 0 | 1 | 2 | 4 | 37 | 2 | 187.5 | 105.4 | 29.980 | 0.849 | 0.143 | 0.517 | 0.481 | 0.574 | 4.120 | 6.230 | 0.890 |
| 1 | 1 | 1 | 1 | 1 | 3 | 26 | 1 | 168.6 | 64.7 | 22.761 | 0.888 | 0.334 | 0.924 | 0.702 | 0.783 | 3.180 | 3.950 | 1.320 |
| 1 | 1 | 1 | 1 | 1 | 3 | 24 | 1 | 163.5 | 65.7 | 24.577 | 1.006 | 0.355 | 0.998 | 0.793 | 0.903 | 3.360 | 4.110 | 1.460 |
| 1 | 1 | 1 | 1 | 1 | 3 | 26 | 1 | 167.0 | 57.0 | 20.438 | 1.006 | 1.066 | 1.598 | 2.038 | 1.209 | 3.480 | 3.480 | 3.710 |
| 1 | 1 | 1 | 2 | 2 | 2 | 60 | 2 | 174.5 | 85.1 | 27.947 | 1.125 | 0.179 | 0.847 | 1.091 | 1.034 | 3.870 | 5.370 | 0.960 |
| 1 | 1 | 1 | 1 | 1 | 3 | 23 | 1 | 169.0 | 51.5 | 18.032 | 1.092 | 0.763 | 1.220 | 1.572 | 1.079 | 3.920 | 4.140 | 3.160 |
| 1 | 0 | 0 | 2 | 2 | 2 | 61 | 2 | 175.6 | 82.6 | 26.787 | 0.991 | 0.091 | 0.447 | 0.570 | 0.549 | 3.420 | 5.410 | 0.490 |
| 1 | 1 | 1 | 2 | 1 | 2 | 63 | 2 | 165.2 | 68.1 | 24.953 | 1.169 | 0.105 | 0.546 | 0.732 | 0.679 | 3.450 | 4.930 | 0.520 |
| 1 | 1 | 1 | 2 | 2 | 1 | 74 | 1 | 158.7 | 69.6 | 27.635 | 0.980 | #NULL! | #NULL! | #NULL! | #NULL! | 1.784 | 2.475 | #NULL! |
| 1 | 1 | 1 | 2 | 1 | 2 | 69 | 2 | 166.5 | 65.3 | 23.555 | 1.210 | 0.370 | 1.020 | 2.517 | 1.089 | 3.400 | 4.080 | 1.510 |
| 1 | 1 | 1 | 1 | 1 | 3 | 25 | 1 | 162.3 | 58.8 | 22.322 | 0.945 | 0.406 | 1.188 | 0.847 | 0.967 | 3.090 | 3.670 | 1.490 |
| 1 | 1 | 1 | 1 | 1 | 3 | 31 | 1 | 163.0 | 59.4 | 22.357 | 1.183 | 0.438 | 1.233 | 1.288 | 1.267 | 3.810 | 4.500 | 1.970 |
| 1 | 0 | 0 | 2 | 1 | 2 | 64 | 2 | 181.0 | 73.4 | 22.405 | 0.885 | 0.078 | 0.408 | 0.488 | 0.475 | 3.170 | 5.270 | 0.410 |
| 1 | 1 | 1 | 2 | 1 | 1 | 72 | 1 | 158.8 | 62.3 | 24.705 | 1.219 | 0.273 | 1.031 | 0.814 | 0.917 | 2.280 | 2.890 | 0.790 |
| 1 | 1 | 1 | 2 | 2 | 1 | 73 | 1 | 178.8 | 84.2 | 26.338 | 1.035 | 0.086 | 0.656 | 0.647 | 0.677 | 2.690 | 3.840 | 0.330 |
| 1 | 0 | 0 | 1 | 2 | 4 | 32 | 2 | 187.0 | 101.7 | 29.083 | 0.819 | 0.106 | 0.436 | 0.338 | 0.495 | 4.060 | 6.510 | 0.690 |
| 1 | 1 | 1 | 1 | 1 | 4 | 22 | 2 | 177.2 | 71.5 | 22.771 | 1.203 | 0.588 | 1.178 | 1.555 | 1.315 | 5.580 | 6.240 | 3.670 |
| 1 | 1 | 1 | 2 | 1 | 2 | 72 | 2 | 187.0 | 82.0 | 23.449 | 0.935 | 0.119 | 0.369 | 0.900 | 0.433 | 3.290 | 5.280 | 0.630 |
| 1 | 1 | 1 | 2 | 2 | 2 | 61 | 2 | 168.0 | 75.0 | 26.573 | 1.147 | 0.149 | 0.954 | 0.911 | 1.114 | 3.590 | 4.820 | 0.720 |
| 1 | 0 | 0 | 2 | 2 | 1 | 75 | 1 | 168.5 | 89.5 | 31.523 | 0.803 | 0.039 | 0.202 | 0.310 | 0.196 | 1.790 | 3.320 | 0.130 |
| 1 | 0 | 0 | 2 | 2 | 1 | 72 | 1 | 159.7 | 69.1 | 27.094 | 1.072 | 0.068 | 0.344 | 0.595 | 0.388 | 2.220 | 3.690 | 0.250 |
| 1 | 1 | 1 | 1 | 1 | 3 | 25 | 1 | 167.0 | 54.5 | 19.542 | 0.991 | 0.316 | 0.933 | 0.711 | 0.849 | 3.440 | 4.210 | 1.330 |
| 1 | 1 | 1 | 1 | 1 | 3 | 21 | 1 | 167.0 | 52.7 | 18.896 | 1.037 | 0.782 | 1.164 | 1.549 | 1.026 | 3.650 | 4.080 | 3.190 |
| 1 | 0 | 0 | 2 | 1 | 1 | 66 | 1 | 158.0 | 49.6 | 19.869 | 0.959 | 0.064 | 0.338 | 0.460 | 0.358 | 2.100 | 3.580 | 0.230 |
| 0 | 0 | 0 | 2 | 1 | 1 | 67 | 1 | 166.5 | 62.7 | 22.617 | 0.752 | 0.240 | 0.373 | 0.720 | 0.371 | 1.730 | 3.540 | 0.850 |
| 1 | 0 | 0 | 1 | 2 | 4 | 31 | 2 | 180.1 | 92.5 | 28.518 | 1.094 | 0.187 | 0.577 | 0.693 | 0.744 | 5.020 | 7.100 | 1.330 |
| 1 | 0 | 0 | 1 | 2 | 4 | 27 | 2 | 188.3 | 97.9 | 27.611 | 0.877 | 0.241 | 0.542 | 0.682 | 0.602 | 4.550 | 6.600 | 1.590 |
| 1 | 1 | 1 | 1 | 1 | 4 | 21 | 2 | 180.0 | 67.8 | 20.926 | 1.058 | 0.432 | 0.882 | 1.068 | 0.956 | 5.080 | 6.180 | 2.670 |
| 1 | 0 | 0 | 2 | 1 | 1 | 66 | 1 | 171.5 | 64.7 | 21.998 | 0.996 | 0.108 | 0.574 | 0.746 | 0.631 | 2.590 | 4.080 | 0.440 |
| 1 | 1 | 1 | 2 | 2 | 2 | 64 | 2 | 175.5 | 79.7 | 25.876 | 0.949 | 0.128 | 0.946 | 0.756 | 1.009 | 3.170 | 4.610 | 0.590 |
| 1 | 1 | 1 | 1 | 1 | 4 | 29 | 2 | 188.0 | 79.9 | 22.606 | 1.174 | 0.524 | 1.000 | 1.700 | 1.214 | 6.000 | 7.140 | 3.740 |
| 1 | 1 | 1 | 2 | 1 | 1 | 72 | 1 | 174.8 | 60.3 | 19.735 | 1.279 | 0.251 | 0.931 | 2.040 | 1.038 | 3.210 | 4.060 | 1.020 |
| 1 | 1 | 1 | 2 | 2 | 2 | 63 | 2 | 179.6 | 83.6 | 25.918 | 0.961 | 0.115 | 0.558 | 0.694 | 0.638 | 3.410 | 5.140 | 0.590 |
| 1 | 1 | 1 | 2 | 2 | 1 | 70 | 1 | 166.7 | 85.0 | 30.588 | 1.163 | 0.097 | 0.566 | 0.813 | 0.650 | 2.710 | 4.010 | 0.390 |
| 1 | 1 | 1 | 1 | 2 | 4 | 25 | 2 | 186.5 | 107.4 | 30.878 | 1.008 | 0.269 | 0.801 | 0.760 | 0.923 | 5.180 | 6.840 | 1.840 |
| 1 | 1 | 1 | 1 | 1 | 3 | 24 | 1 | 169.1 | 55.5 | 19.409 | 0.874 | 0.437 | 0.926 | 0.842 | 0.748 | 3.130 | 3.780 | 1.650 |
| 1 | 1 | 1 | 2 | 1 | 1 | 67 | 1 | 167.0 | 51.6 | 18.502 | 1.014 | 0.287 | 1.123 | 1.609 | 1.033 | 2.930 | 3.590 | 1.030 |
| 1 | 1 | 1 | 1 | 2 | 4 | 20 | 2 | 180.9 | 90.1 | 27.533 | 1.101 | 0.522 | 0.952 | 1.304 | 1.066 | 5.320 | 6.420 | 3.350 |
| 1 | 1 | 1 | 1 | 1 | 3 | 22 | 1 | 169.6 | 61.6 | 21.416 | 1.077 | 0.497 | 1.078 | 1.101 | 1.060 | 3.910 | 4.610 | 2.290 |
| 1 | 1 | 1 | 1 | 2 | 4 | 30 | 2 | 193.0 | 94.8 | 25.450 | 1.002 | 0.482 | 0.904 | 1.370 | 0.967 | 5.390 | 6.450 | 3.110 |
| 1 | 1 | 1 | 1 | 1 | 4 | 25 | 2 | 181.5 | 77.3 | 23.465 | 1.062 | 0.270 | 0.643 | 0.829 | 0.783 | 5.140 | 7.000 | 1.890 |
| 1 | 1 | 1 | 2 | 1 | 1 | 68 | 1 | 171.5 | 62.5 | 21.250 | 1.382 | 0.157 | 1.123 | 1.345 | 1.448 | 3.510 | 4.720 | 0.740 |
| 1 | 1 | 1 | 2 | 1 | 1 | 76 | 1 | 173.3 | 66.8 | 22.242 | 0.923 | 0.148 | 0.380 | 1.140 | 0.360 | 2.150 | 3.320 | 0.490 |
| 1 | 1 | 1 | 2 | 2 | 2 | 67 | 2 | 169.0 | 95.0 | 33.262 | 1.158 | 0.070 | 0.490 | 0.545 | 0.631 | 3.440 | 5.120 | 0.360 |
| 1 | 1 | 1 | 2 | 2 | 1 | 62 | 1 | 162.0 | 67.6 | 25.758 | 1.182 | 0.190 | 0.955 | 1.200 | 1.011 | 2.860 | 3.790 | 0.720 |
| 1 | 1 | 1 | 2 | 2 | 2 | 73 | 2 | 185.5 | 86.5 | 25.138 | 1.222 | 0.109 | 0.887 | 0.985 | 1.210 | 4.180 | 6.030 | 0.660 |
| 1 | 0 | 0 | 2 | 1 | 1 | 69 | 1 | 162.7 | 51.7 | 19.531 | 0.866 | 0.134 | 0.455 | 0.833 | 0.398 | 1.940 | 2.990 | 0.400 |
| 1 | 1 | 1 | 2 | 1 | 1 | 68 | 1 | 159.8 | 61.2 | 23.966 | 1.174 | 0.216 | 0.811 | 1.500 | 0.799 | 2.570 | 3.330 | 0.720 |
| 1 | 1 | 1 | 2 | 2 | 2 | 69 | 2 | 175.5 | 87.6 | 28.441 | 1.120 | 0.073 | 0.553 | 0.582 | 0.707 | 3.540 | 5.320 | 0.390 |
| 1 | 0 | 0 | 2 | 1 | 2 | 68 | 2 | 178.1 | 76.2 | 24.023 | 1.015 | 0.062 | 0.439 | 0.465 | 0.543 | 3.350 | 5.310 | 0.330 |
| 1 | 1 | 1 | 2 | 2 | 1 | 60 | 1 | 167.0 | 81.9 | 29.366 | 0.875 | 0.359 | 0.441 | 1.603 | 0.357 | 2.310 | 3.040 | 1.090 |
| 1 | 1 | 1 | 2 | 1 | 2 | 65 | 2 | 177.2 | 76.1 | 24.236 | 1.175 | 0.266 | 0.874 | 2.108 | 1.160 | 3.960 | 5.150 | 1.370 |
| 1 | 1 | 1 | 1 | 1 | 4 | 28 | 2 | 183.0 | 72.2 | 21.559 | 1.182 | 0.467 | 1.418 | 1.432 | 1.619 | 5.720 | 6.530 | 3.050 |
| 1 | 1 | 1 | 1 | 1 | 4 | 30 | 2 | 184.0 | 79.3 | 23.423 | 1.000 | 0.401 | 1.174 | 1.131 | 1.199 | 4.840 | 5.810 | 2.330 |
| 1 | 1 | 1 | 1 | 2 | 4 | 36 | 2 | 182.5 | 88.1 | 26.451 | 1.211 | 0.351 | 0.969 | 1.385 | 1.257 | 5.560 | 7.070 | 2.480 |
| 1 | 1 | 1 | 1 | 2 | 4 | 34 | 2 | 188.0 | 92.1 | 26.058 | 1.123 | 0.231 | 0.668 | 0.879 | 0.879 | 5.570 | 7.530 | 1.740 |
| 1 | 1 | 1 | 2 | 1 | 2 | 66 | 2 | 192.2 | 90.9 | 24.607 | 0.858 | 0.113 | 0.690 | 0.652 | 0.724 | 3.430 | 5.130 | 0.580 |
| 1 | 0 | 0 | 2 | 1 | 2 | 71 | 2 | 174.9 | 74.5 | 24.354 | 1.163 | 0.077 | 0.420 | 0.710 | 0.586 | 3.560 | 5.690 | 0.440 |
| 1 | 1 | 1 | 1 | 1 | 3 | 39 | 1 | 170.5 | 55.7 | 19.160 | 0.985 | 0.253 | 0.684 | 0.841 | 0.720 | 3.310 | 4.590 | 1.160 |
| 1 | 0 | 0 | 2 | 2 | 2 | 66 | 2 | 180.0 | 99.0 | 30.556 | 1.017 | 0.110 | 0.290 | 0.872 | 0.406 | 3.520 | 6.210 | 0.680 |
| 1 | 1 | 1 | 1 | 1 | 4 | 25 | 2 | 192.0 | 75.9 | 20.589 | 1.302 | 0.548 | 0.969 | 1.583 | 1.321 | 6.080 | 7.140 | 3.910 |
| 1 | 1 | 1 | 1 | 2 | 3 | 26 | 1 | 151.5 | 63.6 | 27.710 | 1.431 | 0.250 | 0.803 | 0.873 | 0.998 | 4.020 | 5.240 | 1.310 |
| 1 | 1 | 1 | 1 | 1 | 4 | 31 | 2 | 185.0 | 78.5 | 22.936 | 0.899 | 0.335 | 0.670 | 1.000 | 0.712 | 4.380 | 6.060 | 2.030 |
| 1 | 1 | 1 | 2 | 1 | 2 | 70 | 2 | 171.5 | 71.6 | 24.344 | 1.118 | 0.341 | 0.673 | 2.452 | 0.754 | 3.320 | 4.460 | 1.520 |
| 1 | 1 | 1 | 1 | 1 | 3 | 20 | 1 | 178.5 | 66.6 | 20.902 | 0.879 | 0.506 | 0.768 | 0.925 | 0.686 | 3.570 | 4.390 | 2.220 |
| 1 | 1 | 1 | 2 | 2 | 2 | 63 | 2 | 174.0 | 79.4 | 26.225 | 1.199 | 0.218 | 1.105 | 1.375 | 1.298 | 3.970 | 5.040 | 1.100 |
| 1 | 1 | 1 | 1 | 1 | 4 | 34 | 2 | 185.0 | 80.9 | 23.638 | 1.165 | 0.245 | 0.644 | 0.632 | 0.829 | 5.220 | 7.220 | 1.770 |

| MEF50 | MEF75 | MEF75MEF75 | FEV1_A | FEV1pred | FEV1_Z | FVC_A | FVCpred | FVC_Z | FEV1FVC | FEV1FVCpred | FEV1FVCZ | Vemax | APC_rest | APC_max | VE_rest | VE_max |
|---|---|---|---|---|---|---|---|---|---|---|---|---|---|---|---|---|
| 5.710 | 8.470 | 0.977 | 4.990 | 4.818 | 0.295 | 6.120 | 5.932 | 0.262 | 0.815 | 0.816 | -0.019 | 103.795 | 31.330 | 293.763 | 11.765 | 103.795 |
| 0.860 | 2.650 | 0.360 | #NULL! | #NULL! | #NULL! | #NULL! | #NULL! | #NULL! | #NULL! | #NULL! | #NULL! | 59.025 | 70.533 | 548.375 | 11.676 | 59.025 |
| 4.360 | 6.180 | 0.969 | 3.610 | 3.572 | 0.090 | 4.190 | 4.224 | -0.064 | 0.862 | 0.851 | 0.168 | 85.134 | 125.460 | 842.458 | 8.644 | 85.134 |
| 1.630 | 2.270 | 0.388 | #NULL! | #NULL! | #NULL! | #NULL! | #NULL! | #NULL! | #NULL! | #NULL! | #NULL! | 62.175 | 123.067 | 441.833 | 8.875 | 62.175 |
| 6.450 | 8.710 | 1.026 | 4.750 | 4.613 | 0.247 | 5.260 | 5.614 | -0.530 | 0.903 | 0.826 | 1.373 | 88.265 | 54.860 | 489.933 | 7.151 | 88.265 |
| 5.030 | 7.870 | 1.261 | 3.050 | 3.423 | -0.916 | 3.580 | 4.065 | -0.971 | 0.852 | 0.847 | 0.077 | 98.591 | 462.673 | 1165.804 | 6.816 | 98.591 |
| 4.760 | 10.120 | 1.584 | 4.190 | 3.572 | 1.496 | 4.770 | 4.123 | 1.247 | 0.878 | 0.874 | 0.082 | 88.343 | 54.897 | 391.854 | 12.536 | 88.343 |
| 3.500 | 4.830 | 0.779 | 3.060 | 3.284 | -0.579 | 3.690 | 3.801 | -0.235 | 0.829 | 0.869 | -0.623 | 54.699 | 345.080 | 969.733 | 9.464 | 54.699 |
| 5.470 | 6.820 | 1.089 | 3.800 | 3.382 | 1.067 | 4.040 | 3.888 | 0.313 | 0.941 | 0.876 | 1.168 | 84.259 | 23.523 | 391.642 | 12.728 | 84.259 |
| 3.170 | 5.230 | 0.976 | 2.790 | 2.525 | 0.728 | 3.860 | 3.205 | 1.334 | 0.723 | 0.793 | -0.998 | 67.550 | 305.763 | 2806.551 | 7.893 | 67.550 |
| 2.700 | 5.460 | 1.073 | 2.770 | 2.216 | 1.578 | 4.120 | 2.859 | 2.530 | 0.672 | 0.781 | -1.371 | 53.783 | 313.693 | 766.009 | 5.550 | 53.783 |
| 5.270 | 6.290 | 1.045 | 3.020 | 3.074 | -0.151 | 3.280 | 3.512 | -0.535 | 0.921 | 0.880 | 0.706 | 81.785 | 250.933 | 676.188 | 8.361 | 81.785 |
| 2.780 | 3.870 | 0.726 | 2.580 | 2.476 | 0.278 | 3.510 | 3.168 | 0.678 | 0.735 | 0.787 | -0.724 | 50.483 | 461.100 | 744.846 | 7.289 | 50.483 |
| 3.220 | 7.270 | 0.837 | 4.120 | 4.847 | -1.203 | 6.230 | 6.041 | 0.255 | 0.661 | 0.807 | -2.206 | 118.905 | 360.510 | 1413.941 | 14.618 | 118.905 |
| 3.650 | 6.760 | 1.055 | 3.180 | 3.530 | -0.837 | 3.950 | 4.140 | -0.369 | 0.805 | 0.859 | -0.821 | 75.955 | 470.563 | 567.473 | 11.233 | 75.955 |
| 4.100 | 6.310 | 1.011 | 3.360 | 3.336 | 0.061 | 4.110 | 3.865 | 0.506 | 0.818 | 0.868 | -0.785 | 68.724 | 70.580 | 323.200 | 8.012 | 68.724 |
| 5.560 | 9.460 | 1.494 | 3.480 | 3.460 | 0.050 | 3.480 | 4.051 | -1.147 | 1.000 | 0.859 | 2.731 | 102.136 | 321.387 | 821.386 | 13.209 | 102.136 |
| 4.550 | 6.000 | 0.820 | 3.870 | 3.442 | 0.870 | 5.370 | 4.438 | 1.440 | 0.721 | 0.777 | -0.806 | 77.842 | 266.457 | 1537.431 | 10.712 | 77.842 |
| 5.050 | 5.890 | 0.917 | 3.920 | 3.593 | 0.782 | 4.140 | 4.165 | -0.048 | 0.947 | 0.869 | 1.423 | 92.606 | 54.870 | 333.500 | 11.427 | 92.606 |
| 2.420 | 5.270 | 0.717 | 3.420 | 3.454 | -0.068 | 5.410 | 4.468 | 1.433 | 0.632 | 0.775 | -1.905 | 105.353 | 211.690 | 1077.758 | 15.436 | 105.353 |
| 2.690 | 8.190 | 1.219 | 3.450 | 2.953 | 1.146 | 4.930 | 3.789 | 2.007 | 0.700 | 0.779 | -1.072 | 95.140 | 421.257 | 2340.729 | 10.346 | 95.140 |
| #NULL! | #NULL! | #NULL! | 1.784 | 1.990 | -0.613 | 2.475 | 2.578 | -0.228 | 0.721 | 0.779 | -0.723 | 59.180 | 415.400 | 1492.198 | 9.356 | 59.180 |
| 4.160 | 5.210 | 0.787 | 3.400 | 2.811 | 1.336 | 4.080 | 3.660 | 0.730 | 0.833 | 0.769 | 0.869 | 79.525 | 141.003 | 1498.321 | 13.058 | 79.525 |
| 4.360 | 5.960 | 0.961 | 3.090 | 3.270 | -0.467 | 3.670 | 3.800 | -0.275 | 0.842 | 0.866 | -0.375 | 81.846 | 84.633 | 432.417 | 7.183 | 81.846 |
| 5.550 | 5.630 | 0.928 | 3.810 | 3.216 | 1.583 | 4.500 | 3.817 | 1.421 | 0.847 | 0.847 | -0.006 | 97.168 | 246.200 | 737.800 | 8.341 | 97.168 |
| 2.150 | 5.360 | 0.709 | 3.170 | 3.577 | -0.739 | 5.270 | 4.682 | 0.833 | 0.602 | 0.767 | -2.117 | 73.965 | 125.477 | 918.609 | 10.939 | 73.965 |
| 2.980 | 4.380 | 0.892 | 2.280 | 2.047 | 0.697 | 2.890 | 2.642 | 0.541 | 0.789 | 0.781 | 0.106 | 27.896 | 2258.510 | 2449.746 | 9.937 | 27.896 |
| 2.520 | 6.870 | 1.242 | 2.690 | 2.598 | 0.215 | 3.840 | 3.416 | 0.705 | 0.701 | 0.770 | -0.877 | 43.907 | 988.167 | 3712.875 | 7.896 | 41.065 |
| 2.840 | 5.250 | 0.596 | 4.060 | 4.962 | -1.472 | 6.510 | 6.111 | 0.544 | 0.624 | 0.817 | -2.728 | 122.545 | 78.403 | 334.611 | 10.367 | 122.545 |
| 7.350 | 10.720 | 1.264 | 5.580 | 4.637 | 1.822 | 6.240 | 5.495 | 1.166 | 0.894 | 0.851 | 0.693 | 119.683 | 78.380 | 675.667 | 14.801 | 119.683 |
| 1.950 | 5.630 | 0.736 | 3.290 | 3.517 | -0.386 | 5.280 | 4.715 | 0.744 | 0.623 | 0.751 | -1.557 | 73.319 | 164.643 | 642.617 | 13.969 | 73.319 |
| 4.600 | 8.350 | 1.205 | 3.590 | 3.131 | 1.017 | 4.820 | 4.015 | 1.363 | 0.745 | 0.780 | -0.504 | 112.946 | 70.573 | 950.033 | 11.439 | 112.946 |
| 0.670 | 2.280 | 0.443 | 1.790 | 2.230 | -1.144 | 3.320 | 2.919 | 0.762 | 0.539 | 0.772 | -2.599 | 60.641 | 619.003 | 1204.963 | 9.466 | 60.641 |
| 1.270 | 4.730 | 0.957 | 2.220 | 2.072 | 0.437 | 3.690 | 2.676 | 2.136 | 0.602 | 0.780 | -2.107 | 48.920 | 211.683 | 2311.350 | 5.834 | 48.920 |
| 3.930 | 7.720 | 1.216 | 3.440 | 3.475 | -0.084 | 4.210 | 4.053 | 0.309 | 0.817 | 0.863 | -0.710 | 63.313 | 70.563 | 479.971 | 10.382 | 63.313 |
| 4.750 | 4.530 | 0.713 | 3.650 | 3.522 | 0.312 | 4.080 | 4.044 | 0.073 | 0.895 | 0.878 | 0.280 | 77.557 | 517.533 | 872.133 | 11.227 | 77.557 |
| 1.210 | 3.830 | 0.760 | 2.100 | 2.190 | -0.263 | 3.580 | 2.793 | 1.710 | 0.587 | 0.789 | -2.465 | 60.410 | 736.773 | 3368.789 | 10.227 | 60.410 |
| 1.320 | 1.800 | 0.341 | 1.730 | 2.416 | -1.773 | 3.540 | 3.110 | 0.838 | 0.489 | 0.783 | -3.336 | 59.720 | 509.487 | 3091.773 | 10.808 | 59.720 |
| 4.100 | 8.410 | 0.994 | 5.020 | 4.592 | 0.778 | 7.100 | 5.601 | 2.227 | 0.707 | 0.824 | -1.761 | 131.842 | 164.610 | 411.413 | 17.337 | 131.842 |
| 3.580 | 6.360 | 0.704 | 4.550 | 5.194 | -1.039 | 6.600 | 6.322 | 0.373 | 0.689 | 0.828 | -1.987 | 122.144 | 533.337 | 911.154 | 12.564 | 122.144 |
| 5.450 | 9.960 | 1.154 | 5.080 | 4.798 | 0.523 | 6.180 | 5.687 | 0.748 | 0.822 | 0.852 | -0.460 | 121.109 | 70.570 | 270.257 | 13.621 | 121.109 |
| 2.340 | 3.300 | 0.603 | 2.590 | 2.605 | -0.038 | 4.080 | 3.362 | 1.302 | 0.635 | 0.782 | -1.880 | 33.667 | 117.603 | 156.727 | 9.017 | 33.667 |
| 4.360 | 5.670 | 0.781 | 3.170 | 3.340 | -0.334 | 4.610 | 4.346 | 0.403 | 0.688 | 0.770 | -1.111 | 117.290 | 703.667 | 9205.875 | 10.506 | 117.290 |
| 7.140 | 10.010 | 1.118 | 6.000 | 5.112 | 1.474 | 7.140 | 6.256 | 1.188 | 0.840 | 0.823 | 0.293 | 105.254 | 133.290 | 989.096 | 15.011 | 105.254 |
| 3.780 | 6.880 | 1.267 | 3.210 | 2.509 | 1.731 | 4.060 | 3.283 | 1.348 | 0.791 | 0.773 | 0.239 | 76.059 | 415.520 | 6217.600 | 11.972 | 76.059 |
| 2.870 | 8.180 | 1.089 | 3.410 | 3.554 | -0.270 | 5.140 | 4.636 | 0.728 | 0.663 | 0.769 | -1.419 | 106.423 | 148.800 | 822.875 | 7.089 | 106.423 |
| 2.270 | 5.350 | 1.025 | 2.710 | 2.330 | 1.023 | 4.010 | 3.016 | 1.904 | 0.676 | 0.779 | -1.310 | 62.446 | 814.767 | 2898.800 | 7.877 | 62.446 |
| 5.480 | 8.190 | 0.911 | 5.180 | 5.143 | 0.061 | 6.840 | 6.211 | 0.866 | 0.757 | 0.835 | -1.161 | 138.489 | 125.450 | 332.933 | 22.111 | 138.489 |
| 3.500 | 5.190 | 0.808 | 3.130 | 3.583 | -1.067 | 3.780 | 4.171 | -0.759 | 0.828 | 0.865 | -0.587 | 81.319 | 107.767 | 333.375 | 5.692 | 81.319 |
| 4.030 | 6.810 | 1.016 | 2.930 | 2.432 | 1.330 | 3.590 | 3.131 | 0.888 | 0.816 | 0.783 | 0.476 | 50.547 | 141.057 | 2570.675 | 11.579 | 50.547 |
| 6.110 | 7.120 | 0.820 | 5.320 | 4.833 | 0.897 | 6.420 | 5.717 | 1.061 | 0.829 | 0.855 | -0.395 | 147.188 | 690.167 | 731.556 | 14.621 | 147.188 |
| 4.970 | 6.970 | 1.082 | 3.910 | 3.631 | 0.660 | 4.610 | 4.195 | 0.789 | 0.848 | 0.873 | -0.397 | 93.485 | 76.867 | 1172.125 | 8.808 | 93.485 |
| 5.830 | 8.480 | 0.922 | 5.390 | 5.386 | 0.007 | 6.450 | 6.642 | -0.244 | 0.836 | 0.817 | 0.317 | 88.456 | 195.987 | 715.229 | 11.551 | 88.456 |
| 4.500 | 7.540 | 0.866 | 5.140 | 4.842 | 0.532 | 7.000 | 5.816 | 1.734 | 0.734 | 0.839 | -1.513 | 148.638 | 54.903 | 971.427 | 15.900 | 148.638 |
| 5.300 | 6.280 | 1.159 | 3.510 | 2.540 | 2.481 | 4.720 | 3.289 | 2.552 | 0.744 | 0.779 | -0.482 | 45.851 | 658.677 | 1312.588 | 7.358 | 45.851 |
| 1.260 | 4.860 | 0.920 | 2.150 | 2.335 | -0.460 | 3.320 | 3.076 | 0.439 | 0.648 | 0.769 | -1.443 | 36.723 | 924.433 | 2066.442 | 9.110 | 36.723 |
| 2.510 | 6.690 | 0.982 | 3.440 | 2.971 | 1.022 | 5.120 | 3.863 | 2.097 | 0.672 | 0.770 | -1.263 | 108.214 | 219.490 | 1429.007 | 11.125 | 108.214 |
| 3.620 | 5.610 | 1.065 | 2.860 | 2.423 | 1.232 | 3.790 | 3.081 | 1.465 | 0.755 | 0.791 | -0.525 | 56.629 | 517.293 | 6358.885 | 5.159 | 56.629 |
| 5.350 | 4.360 | 0.578 | 4.180 | 3.416 | 1.372 | 6.030 | 4.584 | 1.936 | 0.693 | 0.750 | -0.714 | 103.817 | 235.107 | 2408.746 | 11.278 | 103.817 |
| 1.360 | 3.900 | 0.763 | 1.940 | 2.242 | -0.835 | 2.990 | 2.887 | 0.213 | 0.649 | 0.782 | -1.671 | 40.520 | 493.983 | 1048.058 | 6.961 | 40.520 |
| 2.700 | 5.540 | 1.099 | 2.570 | 2.187 | 1.124 | 3.330 | 2.803 | 1.123 | 0.772 | 0.785 | -0.183 | 47.170 | 1261.533 | 3017.995 | 9.275 | 47.170 |
| 2.940 | 5.800 | 0.816 | 3.540 | 3.160 | 0.760 | 5.320 | 4.156 | 1.776 | 0.665 | 0.763 | -1.231 | 73.958 | 148.863 | 382.071 | 10.383 | 73.958 |
| 2.330 | 4.430 | 0.609 | 3.350 | 3.301 | 0.093 | 5.310 | 4.345 | 1.422 | 0.631 | 0.763 | -1.652 | 82.333 | 399.633 | 3515.583 | 14.677 | 82.333 |
| 1.340 | 4.180 | 0.763 | 2.310 | 2.644 | -0.862 | 3.040 | 3.367 | -0.651 | 0.760 | 0.791 | -0.458 | 58.994 | 211.600 | 1097.133 | 11.102 | 58.994 |
| 4.500 | 4.630 | 0.815 | 3.960 | 3.375 | 1.152 | 5.150 | 4.409 | 1.105 | 0.769 | 0.768 | 0.018 | 74.842 | 211.433 | 2761.917 | 8.276 | 74.842 |
| 9.260 | 11.350 | 1.303 | 5.720 | 4.844 | 1.542 | 6.530 | 5.882 | 0.931 | 0.876 | 0.829 | 0.804 | 91.958 | 109.783 | 342.829 | 9.412 | 91.958 |
| 6.820 | 8.960 | 1.029 | 4.840 | 4.844 | -0.007 | 5.810 | 5.919 | -0.155 | 0.833 | 0.823 | 0.162 | 135.068 | 94.050 | 749.313 | 14.628 | 135.068 |
| 6.850 | 6.470 | 0.766 | 5.560 | 4.593 | 1.748 | 7.070 | 5.680 | 1.993 | 0.786 | 0.812 | -0.437 | 134.744 | 86.257 | 546.092 | 13.071 | 134.744 |
| 5.030 | 9.820 | 1.115 | 5.570 | 4.964 | 1.010 | 7.530 | 6.145 | 1.853 | 0.740 | 0.812 | -1.170 | 157.468 | 39.173 | 293.795 | 13.808 | 157.468 |
| 3.540 | 5.190 | 0.640 | 3.430 | 3.998 | -0.898 | 5.130 | 5.317 | -0.230 | 0.669 | 0.756 | -1.165 | 126.693 | 117.613 | 2593.149 | 18.664 | 126.693 |
| 2.390 | 4.840 | 0.689 | 3.560 | 3.066 | 1.002 | 5.690 | 4.049 | 2.523 | 0.626 | 0.760 | -1.635 | 73.704 | 133.270 | 2110.898 | 10.177 | 73.704 |
| 3.140 | 4.020 | 0.657 | 3.310 | 3.365 | -0.134 | 4.590 | 4.122 | 0.900 | 0.721 | 0.822 | -1.517 | 63.835 | 30.733 | 2353.250 | 8.810 | 63.835 |
| 1.800 | 4.580 | 0.616 | 3.520 | 3.456 | 0.119 | 6.210 | 4.538 | 2.391 | 0.567 | 0.764 | -2.433 | 96.934 | 290.003 | 510.189 | 13.772 | 96.934 |
| 6.920 | 8.540 | 1.193 | 6.080 | 5.486 | 0.941 | 7.140 | 6.662 | 0.614 | 0.852 | 0.831 | 0.331 | 149.348 | 54.910 | 338.240 | 17.040 | 149.348 |
| 4.210 | 6.300 | 1.081 | 4.020 | 2.814 | 3.754 | 5.240 | 3.250 | 4.699 | 0.767 | 0.869 | -1.449 | 121.734 | 172.380 | 1756.368 | 7.397 | 121.734 |
| 4.060 | 6.470 | 0.741 | 4.380 | 4.874 | -0.830 | 6.060 | 5.976 | 0.118 | 0.723 | 0.821 | -1.505 | 134.680 | 101.920 | 1052.955 | 16.889 | 134.680 |
| 3.000 | 2.990 | 0.436 | 3.320 | 2.968 | 0.740 | 4.460 | 3.896 | 0.913 | 0.744 | 0.764 | -0.256 | 81.064 | 846.657 | 4758.896 | 14.174 | 81.064 |
| 3.370 | 4.980 | 0.741 | 3.570 | 4.061 | -1.025 | 4.390 | 4.688 | -0.517 | 0.813 | 0.876 | -0.973 | 98.564 | 54.893 | 383.939 | 10.309 | 98.564 |
| 5.570 | 10.720 | 1.489 | 3.970 | 3.313 | 1.353 | 5.040 | 4.295 | 1.160 | 0.788 | 0.773 | 0.213 | 46.656 | 760.740 | 2293.108 | 4.012 | 46.656 |
| 4.650 | 8.060 | 0.933 | 5.220 | 4.790 | 0.740 | 7.220 | 5.911 | 1.821 | 0.723 | 0.815 | -1.443 | 163.902 | 125.473 | 1009.071 | 13.089 | 163.902 |

| APE_rest | APE_max | VT_rest | VT_max | BF_rest | BF_max | Rin5 | Rin20 | R5_minus_R20_in | Xin5 | Xin20 | Rex5 | Rex20 | R5_minus_R20_ex | Xex5 | Xex20 | R5 |
|---|---|---|---|---|---|---|---|---|---|---|---|---|---|---|---|---|
| 368.592 | 30491.027 | 0.802 | 2.929 | 15.136 | 35.676 | #NULL! | #NULL! | #NULL! | #NULL! | #NULL! | #NULL! | #NULL! | #NULL! | #NULL! | #NULL! | #NULL! |
| 823.527 | 32368.022 | 0.700 | 2.162 | 17.031 | 27.494 | 0.345 | 0.265 | 0.080 | -0.170 | -0.015 | 0.550 | 0.255 | 0.295 | -0.350 | -0.090 | 0.450 |
| 1084.487 | 71721.481 | 0.739 | 2.284 | 11.968 | 37.458 | 0.460 | 0.375 | 0.085 | -0.155 | 0.100 | 0.700 | 0.540 | 0.160 | -0.175 | 0.000 | 0.575 |
| 1092.190 | 27471.189 | 0.605 | 1.452 | 15.655 | 43.854 | 0.350 | 0.270 | 0.080 | -0.180 | 0.055 | 0.430 | 0.335 | 0.095 | -0.115 | 0.040 | 0.390 |
| 392.328 | 43243.945 | 0.379 | 1.942 | 19.040 | 45.503 | 0.310 | 0.260 | 0.050 | -0.130 | 0.105 | 0.570 | 0.455 | 0.115 | -0.140 | 0.005 | 0.455 |
| 3153.800 | 114937.431 | 0.461 | 2.106 | 15.131 | 47.040 | 0.420 | 0.360 | 0.060 | -0.135 | 0.080 | 0.515 | 0.435 | 0.080 | -0.130 | 0.080 | 0.470 |
| 688.181 | 34617.647 | 0.904 | 2.322 | 14.652 | 38.223 | 0.400 | 0.310 | 0.090 | -0.130 | 0.080 | 0.435 | 0.345 | 0.090 | -0.135 | 0.070 | 0.420 |
| 3265.814 | 53043.483 | 0.539 | 1.979 | 18.145 | 27.823 | 1.065 | 1.055 | 0.010 | -0.120 | 0.140 | 1.105 | 1.070 | 0.035 | -0.145 | 0.095 | 1.085 |
| 299.416 | 32999.350 | 0.891 | 2.055 | 14.419 | 41.061 | 0.235 | 0.195 | 0.040 | -0.145 | 0.080 | 0.255 | 0.205 | 0.050 | -0.115 | 0.080 | 0.250 |
| 2413.241 | 189582.240 | 0.660 | 1.906 | 12.061 | 35.419 | 0.280 | 0.245 | 0.035 | -0.140 | 0.110 | 0.480 | 0.395 | 0.085 | -0.100 | 0.050 | 0.375 |
| 1740.962 | 41198.069 | 0.409 | 1.779 | 14.485 | 30.764 | 0.260 | 0.170 | 0.090 | -0.130 | 0.070 | 0.355 | 0.235 | 0.120 | -0.120 | 0.100 | 0.315 |
| 2097.991 | 55301.883 | 0.560 | 2.097 | 15.164 | 39.128 | 0.375 | 0.300 | 0.075 | -0.150 | 0.090 | 0.390 | 0.330 | 0.060 | -0.100 | 0.090 | 0.385 |
| 3360.921 | 37601.744 | 0.764 | 1.462 | 9.575 | 34.677 | 0.340 | 0.255 | 0.085 | -0.125 | 0.050 | 0.460 | 0.280 | 0.180 | -0.135 | -0.005 | 0.400 |
| 5269.891 | 168124.814 | 1.717 | 3.295 | 9.150 | 36.102 | 0.315 | 0.200 | 0.115 | -0.105 | 0.030 | 0.395 | 0.230 | 0.165 | -0.140 | 0.015 | 0.355 |
| 5285.830 | 43102.355 | 0.585 | 1.685 | 19.496 | 45.167 | 0.265 | 0.260 | 0.005 | -0.140 | 0.090 | 0.295 | 0.270 | 0.025 | -0.115 | 0.090 | 0.285 |
| 565.521 | 22211.558 | 0.797 | 1.940 | 10.491 | 35.629 | 0.285 | 0.290 | -0.005 | -0.155 | 0.065 | 0.340 | 0.310 | 0.030 | -0.085 | 0.065 | 0.325 |
| 4245.266 | 83892.870 | 0.825 | 1.981 | 16.476 | 51.596 | 0.255 | 0.205 | 0.050 | -0.125 | 0.120 | 0.275 | 0.235 | 0.040 | -0.105 | 0.135 | 0.265 |
| 2854.234 | 119676.705 | 0.939 | 3.026 | 11.719 | 25.873 | 0.235 | 0.170 | 0.065 | -0.125 | 0.040 | 0.290 | 0.205 | 0.085 | -0.105 | 0.030 | 0.260 |
| 626.992 | 30884.092 | 0.530 | 1.526 | 21.706 | 60.875 | 0.265 | 0.185 | 0.080 | -0.125 | 0.055 | 0.330 | 0.240 | 0.090 | -0.100 | 0.040 | 0.305 |
| 3267.621 | 113545.145 | 1.268 | 3.132 | 12.284 | 33.844 | 0.425 | 0.325 | 0.100 | -0.120 | 0.035 | 0.535 | 0.400 | 0.135 | -0.120 | 0.000 | 0.485 |
| 4358.334 | 222696.316 | 0.723 | 2.659 | 14.942 | 36.320 | 0.225 | 0.190 | 0.035 | -0.115 | 0.070 | 0.495 | 0.405 | 0.090 | -0.105 | 0.010 | 0.355 |
| 3886.582 | 88307.955 | 0.732 | 1.161 | 12.936 | 48.378 | 0.310 | 0.250 | 0.060 | -0.115 | 0.090 | 0.570 | 0.450 | 0.120 | -0.095 | 0.070 | 0.435 |
| 1841.158 | 119154.613 | 0.986 | 2.561 | 13.459 | 31.117 | 0.245 | 0.185 | 0.060 | -0.125 | 0.045 | 0.320 | 0.215 | 0.105 | -0.090 | 0.050 | 0.280 |
| 607.963 | 35391.568 | 0.464 | 1.560 | 15.617 | 52.937 | 0.415 | 0.395 | 0.020 | -0.135 | 0.125 | 0.450 | 0.410 | 0.040 | -0.085 | 0.120 | 0.435 |
| 2053.636 | 71690.347 | 0.548 | 2.286 | 16.855 | 42.732 | 0.430 | 0.410 | 0.020 | -0.140 | 0.115 | 0.495 | 0.445 | 0.050 | -0.090 | 0.115 | 0.475 |
| 1372.638 | 67945.173 | 1.359 | 2.002 | 8.637 | 37.305 | 0.265 | 0.200 | 0.065 | -0.110 | 0.030 | 0.295 | 0.225 | 0.070 | -0.085 | 0.025 | 0.280 |
| 22442.142 | 68338.487 | 0.256 | 1.310 | 38.637 | 21.345 | 0.215 | 0.200 | 0.015 | -0.100 | 0.090 | 0.260 | 0.235 | 0.025 | -0.090 | 0.075 | 0.230 |
| 7802.807 | 152468.293 | 0.579 | 1.209 | 13.855 | 33.924 | 0.195 | 0.190 | 0.005 | -0.090 | 0.115 | 0.225 | 0.235 | -0.010 | -0.110 | 0.100 | 0.210 |
| 812.824 | 41004.765 | 0.927 | 3.553 | 11.928 | 34.694 | 0.290 | 0.237 | 0.053 | -0.083 | 0.087 | 0.430 | 0.327 | 0.103 | -0.110 | 0.047 | 0.373 |
| 1160.068 | 80865.545 | 0.956 | 2.844 | 15.591 | 42.154 | 0.220 | 0.135 | 0.085 | -0.085 | 0.060 | 0.285 | 0.170 | 0.115 | -0.105 | 0.065 | 0.255 |
| 2299.838 | 47116.057 | 1.730 | 3.011 | 8.151 | 25.421 | 0.165 | 0.135 | 0.030 | -0.110 | 0.030 | 0.195 | 0.145 | 0.050 | -0.085 | 0.025 | 0.185 |
| 807.323 | 107302.270 | 0.890 | 3.027 | 12.890 | 37.078 | 0.255 | 0.215 | 0.040 | -0.100 | 0.045 | 0.320 | 0.255 | 0.065 | -0.100 | 0.030 | 0.290 |
| 5859.710 | 73069.985 | 0.800 | 1.431 | 12.577 | 42.560 | 0.345 | 0.330 | 0.015 | -0.120 | 0.120 | 0.420 | 0.355 | 0.065 | -0.080 | 0.120 | 0.390 |
| 1235.037 | 113072.207 | 0.503 | 1.797 | 11.661 | 27.195 | 0.320 | 0.250 | 0.070 | -0.125 | 0.085 | 0.320 | 0.260 | 0.060 | -0.065 | 0.080 | 0.320 |
| 732.598 | 30388.402 | 0.767 | 2.018 | 13.862 | 31.379 | 0.740 | 0.680 | 0.060 | -0.100 | 0.210 | 0.700 | 0.620 | 0.080 | -0.090 | 0.200 | 0.720 |
| 5810.174 | 67640.168 | 0.709 | 1.937 | 16.878 | 40.222 | 0.240 | 0.200 | 0.040 | -0.110 | 0.105 | 0.270 | 0.245 | 0.025 | -0.075 | 0.115 | 0.255 |
| 7535.036 | 203509.748 | 0.776 | 1.874 | 13.848 | 32.685 | 0.325 | 0.295 | 0.030 | -0.100 | 0.105 | 0.420 | 0.375 | 0.045 | -0.070 | 0.110 | 0.380 |
| 5506.527 | 184640.590 | 0.632 | 1.992 | 17.474 | 30.187 | 0.235 | 0.205 | 0.030 | -0.105 | 0.070 | 0.270 | 0.235 | 0.035 | -0.080 | 0.080 | 0.255 |
| 2853.872 | 54241.414 | 1.224 | 2.966 | 14.721 | 44.671 | 0.295 | 0.245 | 0.050 | -0.110 | 0.075 | 0.345 | 0.290 | 0.055 | -0.070 | 0.075 | 0.320 |
| 6700.619 | 111292.139 | 1.131 | 3.339 | 11.280 | 36.917 | 0.375 | 0.340 | 0.035 | -0.100 | 0.060 | 0.400 | 0.360 | 0.040 | -0.070 | 0.060 | 0.395 |
| 961.268 | 32730.597 | 1.075 | 2.353 | 13.917 | 51.712 | 0.205 | 0.190 | 0.015 | -0.090 | 0.050 | 0.210 | 0.180 | 0.030 | -0.085 | 0.055 | 0.205 |
| 1060.460 | 5276.439 | 0.717 | 1.630 | 13.406 | 20.871 | 0.290 | 0.250 | 0.040 | -0.085 | 0.105 | 0.365 | 0.305 | 0.060 | -0.085 | 0.095 | 0.325 |
| 7392.971 | 1079756.848 | 0.879 | 2.683 | 12.705 | 43.752 | 0.225 | 0.185 | 0.040 | -0.105 | 0.100 | 0.325 | 0.280 | 0.045 | -0.060 | 0.115 | 0.285 |
| 2000.823 | 104106.191 | 1.271 | 2.979 | 13.268 | 35.442 | 0.360 | 0.305 | 0.055 | -0.090 | 0.105 | 0.385 | 0.320 | 0.065 | -0.070 | 0.075 | 0.370 |
| 4974.672 | 472904.279 | 0.725 | 2.121 | 16.920 | 36.067 | 0.175 | 0.145 | 0.030 | -0.085 | 0.095 | 0.185 | 0.160 | 0.025 | -0.075 | 0.095 | 0.185 |
| 1054.818 | 87573.016 | 0.672 | 2.909 | 10.846 | 36.787 | 0.235 | 0.205 | 0.030 | -0.090 | 0.070 | 0.275 | 0.230 | 0.045 | -0.065 | 0.060 | 0.260 |
| 6417.843 | 181017.058 | 0.753 | 1.999 | 10.478 | 31.114 | 0.295 | 0.275 | 0.020 | -0.100 | 0.105 | 0.355 | 0.325 | 0.030 | -0.050 | 0.095 | 0.325 |
| 2773.815 | 46107.461 | 1.538 | 2.848 | 14.965 | 48.708 | 0.250 | 0.200 | 0.050 | -0.075 | 0.050 | 0.325 | 0.240 | 0.085 | -0.090 | 0.025 | 0.280 |
| 613.417 | 27109.714 | 0.422 | 1.761 | 13.645 | 46.551 | 0.210 | 0.200 | 0.010 | -0.090 | 0.145 | 0.225 | 0.210 | 0.015 | -0.080 | 0.145 | 0.220 |
| 1633.303 | 129940.400 | 0.665 | 1.640 | 17.450 | 31.183 | 0.280 | 0.255 | 0.025 | -0.105 | 0.150 | 0.395 | 0.345 | 0.050 | -0.045 | 0.120 | 0.345 |
| 10090.959 | 107676.112 | 1.064 | 3.053 | 13.920 | 48.493 | 0.305 | 0.260 | 0.045 | -0.090 | 0.110 | 0.320 | 0.260 | 0.060 | -0.060 | 0.110 | 0.310 |
| 677.074 | 109576.484 | 0.574 | 2.281 | 15.431 | 41.342 | 0.290 | 0.270 | 0.020 | -0.060 | 0.155 | 0.325 | 0.315 | 0.010 | -0.095 | 0.160 | 0.310 |
| 2263.803 | 63266.038 | 0.671 | 2.492 | 17.265 | 35.637 | 0.180 | 0.165 | 0.015 | -0.080 | 0.105 | 0.200 | 0.185 | 0.015 | -0.065 | 0.100 | 0.190 |
| 872.967 | 144391.003 | 1.066 | 2.741 | 16.956 | 54.317 | 0.235 | 0.185 | 0.050 | -0.070 | 0.065 | 0.270 | 0.180 | 0.090 | -0.070 | 0.070 | 0.255 |
| 4846.260 | 60182.981 | 0.589 | 1.548 | 13.376 | 30.272 | 0.210 | 0.170 | 0.040 | -0.080 | 0.110 | 0.220 | 0.170 | 0.050 | -0.060 | 0.105 | 0.215 |
| 8421.901 | 75885.260 | 0.896 | 1.471 | 10.280 | 25.320 | 0.235 | 0.190 | 0.045 | -0.070 | 0.055 | 0.295 | 0.190 | 0.105 | -0.065 | 0.045 | 0.265 |
| 2441.750 | 154637.841 | 0.831 | 2.952 | 13.595 | 36.689 | 0.245 | 0.190 | 0.055 | -0.105 | 0.090 | 0.330 | 0.255 | 0.075 | -0.045 | 0.100 | 0.295 |
| 2668.942 | 360097.769 | 0.552 | 2.248 | 10.061 | 25.345 | 0.250 | 0.255 | -0.005 | -0.080 | 0.145 | 0.300 | 0.280 | 0.020 | -0.060 | 0.130 | 0.275 |
| 2651.482 | 250069.400 | 0.890 | 3.234 | 13.164 | 32.262 | 0.305 | 0.235 | 0.070 | -0.080 | 0.100 | 0.370 | 0.310 | 0.060 | -0.045 | 0.120 | 0.335 |
| 3438.479 | 42466.984 | 0.834 | 1.491 | 8.725 | 27.672 | 0.225 | 0.180 | 0.045 | -0.075 | 0.065 | 0.220 | 0.175 | 0.045 | -0.045 | 0.055 | 0.225 |
| 11700.197 | 142357.420 | 0.826 | 1.795 | 11.544 | 26.252 | 0.310 | 0.275 | 0.035 | -0.060 | 0.105 | 0.460 | 0.425 | 0.035 | -0.065 | 0.135 | 0.400 |
| 1545.622 | 28257.314 | 0.743 | 2.443 | 14.309 | 30.374 | 0.325 | 0.250 | 0.075 | -0.075 | 0.110 | 0.370 | 0.305 | 0.065 | -0.050 | 0.115 | 0.345 |
| 5865.552 | 289448.893 | 1.159 | 3.281 | 13.267 | 25.424 | 0.175 | 0.140 | 0.035 | -0.075 | 0.075 | 0.190 | 0.140 | 0.050 | -0.060 | 0.060 | 0.180 |
| 2349.178 | 64724.180 | 0.754 | 1.654 | 15.398 | 35.718 | 0.355 | 0.315 | 0.040 | -0.085 | 0.175 | 0.400 | 0.370 | 0.030 | -0.045 | 0.190 | 0.380 |
| 1749.795 | 206706.112 | 0.575 | 2.280 | 15.560 | 32.997 | 0.140 | 0.110 | 0.030 | -0.075 | 0.060 | 0.175 | 0.130 | 0.045 | -0.045 | 0.065 | 0.160 |
| 1033.289 | 31525.764 | 0.796 | 2.740 | 12.781 | 33.895 | 0.250 | 0.220 | 0.030 | -0.070 | 0.140 | 0.300 | 0.250 | 0.050 | -0.050 | 0.160 | 0.280 |
| 1375.733 | 101208.342 | 0.982 | 2.735 | 16.421 | 49.513 | 0.285 | 0.245 | 0.040 | -0.060 | 0.130 | 0.335 | 0.295 | 0.040 | -0.060 | 0.105 | 0.310 |
| 1127.419 | 73582.489 | 0.708 | 3.233 | 19.259 | 41.725 | 0.205 | 0.170 | 0.035 | -0.080 | 0.065 | 0.225 | 0.175 | 0.050 | -0.040 | 0.070 | 0.220 |
| 540.898 | 46263.305 | 0.716 | 3.136 | 19.474 | 50.337 | 0.195 | 0.160 | 0.035 | -0.070 | 0.090 | 0.205 | 0.170 | 0.035 | -0.050 | 0.090 | 0.205 |
| 2195.096 | 328533.202 | 1.028 | 3.273 | 18.451 | 38.775 | 0.165 | 0.145 | 0.020 | -0.075 | 0.050 | 0.180 | 0.150 | 0.030 | -0.040 | 0.055 | 0.175 |
| 1356.299 | 155581.532 | 0.756 | 2.748 | 13.802 | 27.082 | 0.135 | 0.130 | 0.005 | -0.060 | 0.065 | 0.160 | 0.145 | 0.015 | -0.045 | 0.065 | 0.150 |
| 270.751 | 150220.515 | 0.726 | 2.623 | 12.755 | 24.364 | 0.370 | 0.390 | -0.020 | -0.070 | 0.200 | 0.555 | 0.560 | -0.005 | -0.030 | 0.135 | 0.450 |
| 3994.021 | 49454.730 | 1.017 | 3.427 | 14.190 | 28.481 | 0.270 | 0.210 | 0.060 | -0.055 | 0.045 | 0.310 | 0.245 | 0.065 | -0.045 | 0.035 | 0.290 |
| 935.676 | 50515.343 | 0.965 | 2.878 | 17.815 | 52.049 | 0.195 | 0.155 | 0.040 | -0.065 | 0.065 | 0.200 | 0.170 | 0.030 | -0.035 | 0.075 | 0.195 |
| 1275.124 | 213809.535 | 0.601 | 2.486 | 12.534 | 49.064 | 0.290 | 0.245 | 0.045 | -0.060 | 0.135 | 0.300 | 0.270 | 0.030 | -0.040 | 0.150 | 0.295 |
| 1721.322 | 141811.966 | 0.684 | 2.526 | 24.606 | 53.317 | 0.233 | 0.200 | 0.033 | -0.060 | 0.070 | 0.257 | 0.217 | 0.040 | -0.030 | 0.077 | 0.250 |
| 12000.546 | 385773.710 | 1.015 | 2.946 | 15.002 | 27.503 | 0.315 | 0.295 | 0.020 | -0.060 | 0.115 | 0.330 | 0.290 | 0.040 | -0.030 | 0.105 | 0.325 |
| 565.879 | 37842.445 | 0.593 | 2.028 | 17.674 | 48.806 | 0.250 | 0.280 | -0.030 | -0.050 | 0.240 | 0.270 | 0.295 | -0.025 | -0.030 | 0.240 | 0.260 |
| 3052.319 | 106986.922 | 0.374 | 1.318 | 10.925 | 35.778 | 0.255 | 0.195 | 0.060 | -0.055 | 0.060 | 0.245 | 0.190 | 0.055 | -0.035 | 0.060 | 0.250 |
| 1642.259 | 165388.491 | 1.283 | 3.945 | 10.737 | 41.881 | 0.245 | 0.215 | 0.030 | -0.035 | 0.060 | 0.240 | 0.215 | 0.025 | -0.025 | 0.065 | 0.240 |

| R20 | R5_minus_R20 | X5 | X20 | Z5 | Rzentral | Rperipher | AX | Resonanzfrequenz | R5_in_mean | R5_ex_mean | R5_in_max | R5_ex_max | R5_inex_mean | R20_in_mean | R20_ex_mean | R20_in_max | R20_ex_max |
|---|---|---|---|---|---|---|---|---|---|---|---|---|---|---|---|---|---|
| #NULL! | #NULL! | #NULL! | #NULL! | #NULL! | #NULL! | #NULL! | #NULL! | #NULL! | 0.224 | 0.506 | 0.462 | 1.839 | 0.365 | 0.212 | 0.352 | 0.475 | 0.870 |
| 0.265 | 0.185 | -0.245 | -0.060 | 0.515 | 0.230 | 0.475 | 2.095 | 25.240 | 0.463 | 1.610 | 1.357 | 3.932 | 1.036 | 0.592 | 0.654 | 1.167 | 1.097 |
| 0.465 | 0.110 | -0.165 | 0.060 | 0.595 | 0.320 | 0.425 | 0.610 | 13.925 | 1.290 | 2.245 | 2.602 | 7.946 | 1.768 | 0.792 | 0.947 | 1.000 | 1.155 |
| 0.305 | 0.085 | -0.150 | 0.050 | 0.420 | 0.230 | 0.400 | 0.510 | 13.315 | 0.696 | 1.252 | 2.402 | 4.966 | 0.974 | 0.293 | 0.618 | 0.707 | 0.819 |
| 0.380 | 0.075 | -0.135 | 0.055 | 0.475 | 0.270 | 0.300 | 0.450 | 13.360 | 0.277 | 0.825 | 0.516 | 2.753 | 0.551 | 0.241 | 0.559 | 0.448 | 0.922 |
| 0.400 | 0.070 | -0.135 | 0.080 | 0.490 | 0.315 | 0.275 | 0.365 | 12.860 | 0.346 | 1.078 | 0.998 | 2.865 | 0.712 | 0.290 | 0.649 | 0.784 | 1.255 |
| 0.330 | 0.090 | -0.135 | 0.075 | 0.440 | 0.245 | 0.325 | 0.425 | 13.065 | 0.287 | 0.523 | 0.555 | 1.196 | 0.405 | 0.260 | 0.398 | 0.479 | 0.671 |
| 1.060 | 0.025 | -0.135 | 0.120 | 1.095 | 0.645 | 0.325 | 0.260 | 9.375 | 1.096 | 1.099 | 7.900 | 2.022 | 1.097 | 0.896 | 1.142 | 1.431 | 3.347 |
| 0.200 | 0.050 | -0.130 | 0.080 | 0.280 | 0.090 | 0.150 | 0.300 | 11.400 | 0.155 | 0.512 | 0.373 | 1.989 | 0.334 | 0.119 | 0.275 | 0.229 | 0.535 |
| 0.325 | 0.050 | -0.130 | 0.085 | 0.400 | 0.245 | 0.300 | 0.355 | 12.060 | 0.215 | 1.183 | 0.513 | 3.014 | 0.699 | 0.175 | 0.582 | 0.492 | 1.022 |
| 0.210 | 0.105 | -0.130 | 0.090 | 0.340 | 0.135 | 0.300 | 0.535 | 13.860 | 0.217 | 1.360 | 0.528 | 4.202 | 0.788 | 0.173 | 0.687 | 0.335 | 1.433 |
| 0.320 | 0.065 | -0.130 | 0.090 | 0.405 | 0.245 | 0.300 | 0.360 | 12.225 | 0.252 | 0.737 | 0.802 | 1.956 | 0.494 | 0.224 | 0.557 | 0.601 | 0.926 |
| 0.270 | 0.130 | -0.130 | 0.020 | 0.425 | 0.135 | 0.275 | 0.780 | 17.575 | 0.232 | 1.507 | 0.565 | 3.074 | 0.870 | 0.223 | 0.731 | 0.534 | 0.954 |
| 0.220 | 0.135 | -0.125 | 0.025 | 0.375 | 0.050 | 0.250 | 0.765 | 17.715 | 0.214 | 0.751 | 0.617 | 3.321 | 0.483 | 0.160 | 0.395 | 0.337 | 0.794 |
| 0.265 | 0.020 | -0.125 | 0.095 | 0.310 | 0.195 | 0.300 | 0.255 | 9.690 | 0.302 | 1.312 | 0.876 | 3.601 | 0.807 | 0.270 | 0.515 | 0.556 | 0.785 |
| 0.300 | 0.025 | -0.120 | 0.065 | 0.340 | 0.225 | 0.275 | 0.270 | 10.460 | 0.278 | 0.753 | 0.541 | 1.714 | 0.516 | 0.246 | 0.444 | 0.435 | 0.550 |
| 0.220 | 0.045 | -0.115 | 0.125 | 0.290 | 0.185 | 0.275 | 0.220 | 9.385 | 0.276 | 0.645 | 0.911 | 2.236 | 0.461 | 0.232 | 0.486 | 0.585 | 0.909 |
| 0.185 | 0.075 | -0.115 | 0.035 | 0.285 | 0.130 | 0.250 | 0.455 | 14.995 | 0.225 | 0.929 | 0.595 | 3.350 | 0.577 | 0.175 | 0.578 | 0.399 | 1.047 |
| 0.210 | 0.095 | -0.115 | 0.045 | 0.325 | 0.225 | 0.275 | 0.325 | 12.110 | 0.299 | 0.607 | 0.610 | 1.616 | 0.453 | 0.208 | 0.382 | 0.354 | 0.597 |
| 0.365 | 0.120 | -0.115 | 0.020 | 0.495 | 0.270 | 0.275 | 0.680 | 18.275 | 0.293 | 2.424 | 0.945 | 12.829 | 1.359 | 0.241 | 0.861 | 0.776 | 1.560 |
| 0.305 | 0.050 | -0.115 | 0.045 | 0.380 | 0.235 | 0.250 | 0.340 | 13.805 | 0.238 | 1.820 | 0.701 | 7.204 | 1.029 | 0.173 | 0.621 | 0.422 | 0.904 |
| 0.350 | 0.085 | -0.110 | 0.065 | 0.450 | 0.255 | 0.275 | 0.345 | 12.980 | 0.880 | 1.638 | 2.076 | 7.723 | 1.259 | 0.596 | 0.938 | 0.875 | 1.868 |
| 0.200 | 0.080 | -0.110 | 0.045 | 0.300 | 0.050 | 0.275 | 0.415 | 14.385 | 0.293 | 1.098 | 0.426 | 3.145 | 0.696 | 0.271 | 0.710 | 0.407 | 1.336 |
| 0.400 | 0.035 | -0.110 | 0.125 | 0.450 | 0.260 | 0.250 | 0.215 | 9.465 | 0.262 | 1.274 | 0.616 | 2.307 | 0.768 | 0.375 | 0.548 | 0.605 | 0.670 |
| 0.430 | 0.045 | -0.110 | 0.115 | 0.485 | 0.315 | 0.250 | 0.200 | 9.155 | 0.776 | 2.656 | 2.586 | 12.239 | 1.716 | 0.849 | 1.768 | 3.925 | 14.486 |
| 0.215 | 0.065 | -0.105 | 0.025 | 0.300 | 0.070 | 0.225 | 0.415 | 15.655 | 0.340 | 0.662 | 0.709 | 2.304 | 0.501 | 0.245 | 0.346 | 0.443 | 0.740 |
| 0.220 | 0.010 | -0.100 | 0.085 | 0.250 | 0.135 | 0.175 | 0.185 | 9.560 | 0.310 | 1.038 | 1.260 | 3.873 | 0.674 | 0.214 | 0.445 | 0.398 | 0.672 |
| 0.210 | 0.000 | -0.100 | 0.105 | 0.235 | 0.100 | 0.100 | 0.160 | 8.655 | 0.266 | 1.318 | 0.827 | 3.939 | 0.792 | 0.297 | 0.704 | 0.594 | 1.038 |
| 0.297 | 0.077 | -0.097 | 0.063 | 0.387 | 0.203 | 0.217 | 0.333 | 12.870 | 0.194 | 0.798 | 1.407 | 2.269 | 0.496 | 0.167 | 0.517 | 0.666 | 1.314 |
| 0.150 | 0.105 | -0.095 | 0.065 | 0.275 | 0.120 | 0.200 | 0.375 | 14.635 | 0.200 | 0.616 | 0.356 | 2.942 | 0.408 | 0.146 | 0.285 | 0.253 | 0.607 |
| 0.135 | 0.050 | -0.095 | 0.025 | 0.205 | 0.055 | 0.075 | 0.305 | 13.670 | 0.183 | 1.188 | 0.453 | 10.542 | 0.685 | 0.150 | 0.466 | 0.365 | 0.949 |
| 0.235 | 0.055 | -0.095 | 0.035 | 0.310 | 0.050 | 0.250 | 0.290 | 14.470 | 0.293 | 0.899 | 0.826 | 2.767 | 0.596 | 0.241 | 0.448 | 0.615 | 0.868 |
| 0.340 | 0.050 | -0.095 | 0.120 | 0.400 | 0.240 | 0.250 | 0.170 | 8.825 | 0.490 | 1.122 | 1.586 | 4.096 | 0.806 | 0.333 | 0.565 | 0.554 | 0.811 |
| 0.255 | 0.065 | -0.095 | 0.085 | 0.335 | 0.245 | 0.250 | 0.240 | 12.190 | 0.721 | 1.476 | 1.916 | 7.478 | 1.098 | 0.519 | 0.624 | 0.915 | 1.368 |
| 0.640 | 0.080 | -0.090 | 0.210 | 0.720 | 0.510 | 0.250 | 0.180 | 8.710 | 0.776 | 0.961 | 1.414 | 1.883 | 0.869 | 0.805 | 0.905 | 1.217 | 1.388 |
| 0.225 | 0.030 | -0.090 | 0.110 | 0.270 | 0.120 | 0.200 | 0.165 | 9.065 | 0.197 | 0.699 | 0.412 | 5.027 | 0.448 | 0.180 | 0.426 | 0.388 | 1.125 |
| 0.340 | 0.040 | -0.090 | 0.105 | 0.385 | 0.195 | 0.250 | 0.120 | 8.000 | 0.961 | 1.901 | 1.621 | 5.378 | 1.431 | 0.581 | 0.689 | 0.804 | 1.160 |
| 0.220 | 0.035 | -0.090 | 0.075 | 0.270 | 0.180 | 0.150 | 0.180 | 9.785 | 0.342 | 1.853 | 0.656 | 3.531 | 1.098 | 0.295 | 0.728 | 0.440 | 1.072 |
| 0.270 | 0.050 | -0.090 | 0.075 | 0.330 | 0.230 | 0.250 | 0.190 | 10.390 | 0.386 | 0.996 | 0.856 | 3.100 | 0.691 | 0.335 | 0.416 | 0.502 | 0.614 |
| 0.350 | 0.045 | -0.085 | 0.060 | 0.405 | 0.255 | 0.250 | 0.175 | 10.955 | 0.641 | 0.808 | 1.663 | 2.631 | 0.724 | 0.415 | 0.506 | 0.663 | 1.093 |
| 0.185 | 0.020 | -0.085 | 0.055 | 0.225 | 0.135 | 0.100 | 0.175 | 9.910 | 0.190 | 0.645 | 0.393 | 6.946 | 0.417 | 0.202 | 0.453 | 1.431 | 1.675 |
| 0.275 | 0.050 | -0.085 | 0.105 | 0.335 | 0.235 | 0.250 | 0.135 | 8.690 | 0.325 | 0.653 | 0.626 | 1.374 | 0.489 | 0.236 | 0.354 | 0.380 | 0.504 |
| 0.240 | 0.045 | -0.085 | 0.110 | 0.295 | 0.160 | 0.200 | 0.135 | 8.690 | 0.489 | 1.344 | 1.116 | 9.067 | 0.917 | 0.503 | 0.702 | 1.036 | 2.060 |
| 0.310 | 0.060 | -0.080 | 0.090 | 0.380 | 0.240 | 0.250 | 0.165 | 9.490 | 0.316 | 0.552 | 0.859 | 2.298 | 0.434 | 0.324 | 0.375 | 0.862 | 0.985 |
| 0.155 | 0.030 | -0.080 | 0.095 | 0.195 | 0.070 | 0.075 | 0.130 | 8.765 | 0.285 | 0.364 | 0.640 | 0.668 | 0.324 | 0.207 | 0.236 | 0.330 | 0.336 |
| 0.215 | 0.045 | -0.080 | 0.060 | 0.270 | 0.090 | 0.150 | 0.160 | 10.145 | 0.182 | 1.383 | 0.381 | 6.826 | 0.782 | 0.217 | 0.558 | 0.523 | 0.761 |
| 0.295 | 0.030 | -0.080 | 0.100 | 0.335 | 0.180 | 0.200 | 0.140 | 9.050 | 0.204 | 1.071 | 0.410 | 4.974 | 0.638 | 0.212 | 0.654 | 0.378 | 1.330 |
| 0.215 | 0.065 | -0.080 | 0.040 | 0.295 | 0.095 | 0.175 | 0.295 | 14.265 | 0.486 | 0.833 | 2.550 | 2.455 | 0.659 | 0.329 | 0.536 | 1.099 | 0.987 |
| 0.210 | 0.010 | -0.080 | 0.145 | 0.235 | 0.135 | 0.100 | 0.130 | 8.465 | 0.232 | 0.604 | 0.456 | 5.247 | 0.418 | 0.201 | 0.496 | 0.373 | 1.082 |
| 0.305 | 0.040 | -0.075 | 0.135 | 0.355 | 0.185 | 0.175 | 0.140 | 8.940 | 0.326 | 1.506 | 0.783 | 4.920 | 0.916 | 0.292 | 0.647 | 0.640 | 1.136 |
| 0.255 | 0.055 | -0.075 | 0.110 | 0.320 | 0.215 | 0.250 | 0.130 | 8.720 | 0.341 | 1.008 | 1.885 | 6.958 | 0.675 | 0.344 | 0.631 | 0.960 | 1.615 |
| 0.295 | 0.015 | -0.075 | 0.160 | 0.315 | 0.175 | 0.200 | 0.095 | 7.830 | 0.179 | 0.746 | 0.352 | 1.938 | 0.462 | 0.273 | 0.800 | 0.664 | 1.437 |
| 0.180 | 0.010 | -0.070 | 0.100 | 0.200 | 0.110 | 0.075 | 0.105 | 8.350 | 0.159 | 0.928 | 0.560 | 6.463 | 0.543 | 0.150 | 0.480 | 0.352 | 1.619 |
| 0.185 | 0.070 | -0.070 | 0.065 | 0.265 | 0.065 | 0.150 | 0.195 | 11.505 | 0.272 | 0.819 | 0.592 | 2.817 | 0.545 | 0.235 | 0.642 | 0.709 | 1.276 |
| 0.175 | 0.040 | -0.070 | 0.110 | 0.225 | 0.095 | 0.100 | 0.120 | 8.940 | 0.265 | 0.857 | 0.621 | 2.115 | 0.561 | 0.244 | 0.350 | 0.616 | 0.521 |
| 0.190 | 0.075 | -0.070 | 0.045 | 0.275 | 0.095 | 0.150 | 0.195 | 12.210 | 0.670 | 1.277 | 1.154 | 3.769 | 0.974 | 0.431 | 0.585 | 0.573 | 0.791 |
| 0.225 | 0.070 | -0.070 | 0.090 | 0.305 | 0.105 | 0.150 | 0.160 | 10.860 | 0.263 | 1.337 | 0.933 | 4.535 | 0.800 | 0.208 | 0.792 | 0.626 | 1.176 |
| 0.270 | 0.005 | -0.070 | 0.140 | 0.285 | 0.140 | 0.200 | 0.105 | 8.195 | 0.268 | 0.686 | 0.755 | 1.327 | 0.477 | 0.210 | 0.370 | 0.458 | 0.542 |
| 0.275 | 0.060 | -0.065 | 0.110 | 0.345 | 0.225 | 0.250 | 0.120 | 8.845 | 0.255 | 0.695 | 0.441 | 2.464 | 0.475 | 0.288 | 0.567 | 1.080 | 0.874 |
| 0.175 | 0.050 | -0.065 | 0.065 | 0.235 | 0.110 | 0.075 | 0.145 | 10.155 | 0.242 | 0.466 | 0.446 | 0.878 | 0.354 | 0.196 | 0.324 | 0.307 | 0.469 |
| 0.365 | 0.035 | -0.065 | 0.125 | 0.405 | 0.190 | 0.200 | 0.095 | 8.275 | 0.337 | 1.277 | 0.706 | 3.230 | 0.807 | 0.342 | 0.687 | 0.507 | 1.016 |
| 0.280 | 0.065 | -0.065 | 0.115 | 0.350 | 0.180 | 0.225 | 0.150 | 9.800 | 0.304 | 1.256 | 1.436 | 3.180 | 0.780 | 0.263 | 0.789 | 0.806 | 1.307 |
| 0.140 | 0.040 | -0.065 | 0.070 | 0.190 | 0.075 | 0.100 | 0.115 | 9.055 | 0.194 | 0.632 | 0.498 | 2.530 | 0.413 | 0.211 | 0.360 | 0.510 | 0.790 |
| 0.340 | 0.040 | -0.065 | 0.185 | 0.385 | 0.230 | 0.250 | 0.090 | 7.770 | 0.573 | 1.128 | 1.295 | 2.243 | 0.851 | 0.729 | 0.830 | 1.227 | 1.177 |
| 0.120 | 0.040 | -0.065 | 0.065 | 0.175 | 0.020 | 0.100 | 0.135 | 10.520 | 0.131 | 0.914 | 0.424 | 3.661 | 0.523 | 0.102 | 0.444 | 0.379 | 1.268 |
| 0.240 | 0.040 | -0.060 | 0.150 | 0.280 | 0.080 | 0.150 | 0.080 | 7.930 | 0.439 | 0.838 | 0.969 | 1.957 | 0.638 | 0.455 | 0.596 | 1.211 | 0.976 |
| 0.270 | 0.040 | -0.060 | 0.115 | 0.315 | 0.115 | 0.150 | 0.095 | 8.280 | 0.406 | 0.637 | 0.854 | 3.504 | 0.522 | 0.399 | 0.471 | 0.917 | 1.606 |
| 0.175 | 0.045 | -0.060 | 0.070 | 0.225 | 0.075 | 0.100 | 0.110 | 9.260 | 0.361 | 1.095 | 1.239 | 3.152 | 0.728 | 0.247 | 0.513 | 0.411 | 0.770 |
| 0.165 | 0.040 | -0.055 | 0.090 | 0.215 | 0.115 | 0.100 | 0.105 | 8.940 | 0.232 | 1.028 | 0.529 | 4.307 | 0.630 | 0.216 | 0.498 | 0.543 | 0.836 |
| 0.150 | 0.025 | -0.055 | 0.055 | 0.185 | 0.025 | 0.100 | 0.100 | 9.110 | 0.384 | 1.134 | 1.420 | 3.467 | 0.759 | 0.154 | 0.441 | 0.373 | 0.616 |
| 0.135 | 0.015 | -0.055 | 0.065 | 0.155 | 0.020 | 0.075 | 0.070 | 8.170 | 0.700 | 1.397 | 1.075 | 8.353 | 1.048 | 0.341 | 0.519 | 0.529 | 0.830 |
| 0.470 | -0.020 | -0.055 | 0.175 | 0.455 | 0.225 | 0.250 | 0.060 | 7.260 | 0.396 | 1.201 | 1.015 | 3.233 | 0.798 | 0.412 | 0.573 | 0.632 | 0.880 |
| 0.225 | 0.065 | -0.050 | 0.040 | 0.295 | 0.105 | 0.150 | 0.160 | 13.080 | 0.188 | 0.547 | 0.401 | 2.111 | 0.367 | 0.154 | 0.326 | 0.281 | 0.661 |
| 0.165 | 0.030 | -0.050 | 0.070 | 0.205 | 0.085 | 0.100 | 0.075 | 8.165 | 0.538 | 0.960 | 0.988 | 3.930 | 0.749 | 0.233 | 0.576 | 0.492 | 1.188 |
| 0.265 | 0.030 | -0.050 | 0.140 | 0.300 | 0.090 | 0.150 | 0.095 | 8.615 | 0.400 | 1.909 | 0.745 | 5.282 | 1.155 | 0.437 | 0.759 | 0.615 | 1.108 |
| 0.210 | 0.040 | -0.047 | 0.077 | 0.250 | 0.090 | 0.100 | 0.067 | 8.157 | 0.213 | 1.044 | 0.454 | 4.939 | 0.629 | 0.249 | 0.484 | 0.553 | 0.924 |
| 0.295 | 0.030 | -0.040 | 0.110 | 0.325 | 0.110 | 0.175 | 0.050 | 7.510 | 0.348 | 0.858 | 0.698 | 3.256 | 0.603 | 0.341 | 0.497 | 0.653 | 0.777 |
| 0.290 | -0.030 | -0.040 | 0.245 | 0.265 | 0.095 | 0.100 | 0.020 | 6.180 | 0.233 | 0.440 | 0.464 | 0.917 | 0.336 | 0.262 | 0.506 | 0.595 | 0.966 |
| 0.190 | 0.060 | -0.035 | 0.060 | 0.250 | 0.060 | 0.150 | 0.120 | 10.660 | 0.240 | 1.008 | 0.561 | 5.490 | 0.624 | 0.198 | 0.545 | 0.361 | 1.479 |
| 0.215 | 0.025 | -0.030 | 0.060 | 0.245 | 0.095 | 0.100 | 0.040 | 7.820 | 0.385 | 0.987 | 2.143 | 4.840 | 0.686 | 0.223 | 0.350 | 0.631 | 0.513 |

| R20_inex_mean | R5_minus_R20_inex_n | VC | R5_ex_50 | R5_ex_60 | R5_ex_75 | R5_ex_85 | R5_ex_95 | R20_ex_75 | R20_ex_95 | Raucher1_never0 | ERVsoll | ERV | rel_ERV | Temperatu | Druck | rH |
|---|---|---|---|---|---|---|---|---|---|---|---|---|---|---|---|---|
| 0.282 | 0.083 | #NULL! | 0.672 | 0.721 | 0.801 | 0.866 | 0.947 | 0.439 | 0.461 | 0.000 | 1.620 | 2.090 | 1.290 | 20.6 | 956 | 22.6 |
| 0.623 | 0.413 | #NULL! | 1.820 | 1.844 | 1.872 | 1.951 | 1.947 | 0.715 | 0.720 | 1.000 | 1.100 | 0.090 | 0.082 | 23.8 | 943 | 35.2 |
| 0.870 | 0.898 | #NULL! | 2.588 | 2.651 | 2.773 | 2.828 | 3.173 | 0.985 | 0.988 | 1.000 | 1.310 | 1.680 | 1.282 | 25.0 | 954 | 43.5 |
| 0.456 | 0.518 | #NULL! | 1.419 | 1.431 | 1.450 | 1.466 | 1.537 | 0.659 | 0.661 | 0.000 | 1.290 | 1.220 | 0.946 | 23.6 | 952 | 46.7 |
| 0.400 | 0.151 | #NULL! | 0.789 | 0.780 | 0.767 | 0.730 | 0.760 | 0.546 | 0.554 | 1.000 | 1.600 | 2.030 | 1.269 | 23.9 | 955 | 21.3 |
| 0.470 | 0.242 | #NULL! | 1.396 | 1.469 | 1.569 | 1.623 | 1.579 | 0.838 | 0.876 | 0.000 | 1.270 | 0.650 | 0.512 | 24.8 | 948 | 13.3 |
| 0.329 | 0.076 | #NULL! | 0.624 | 0.633 | 0.637 | 0.659 | 0.699 | 0.455 | 0.461 | 0.000 | 1.390 | 1.490 | 1.072 | 24.7 | 954 | 14.2 |
| 1.019 | 0.079 | #NULL! | 1.182 | 1.188 | 1.209 | 1.239 | 1.245 | 1.026 | 1.015 | 1.000 | 1.340 | 1.470 | 1.097 | 25.1 | 953 | 10.4 |
| 0.197 | 0.136 | #NULL! | 0.591 | 0.592 | 0.607 | 0.612 | 0.620 | 0.291 | 0.291 | 0.000 | 1.380 | 0.920 | 0.667 | 21.5 | 942 | 24.7 |
| 0.379 | 0.320 | #NULL! | 1.616 | 1.668 | 1.780 | 1.920 | 1.556 | 0.731 | 0.744 | 0.000 | 0.800 | 0.630 | 0.788 | 25.4 | 955 | 11.2 |
| 0.430 | 0.358 | #NULL! | 1.468 | 1.499 | 1.589 | 1.685 | 0.717 | 0.723 | 0.733 | 1.000 | 0.650 | 1.470 | 2.262 | 25.9 | 959 | 21.0 |
| 0.390 | 0.104 | #NULL! | 0.889 | 0.915 | 0.982 | 1.040 | 1.111 | 0.650 | 0.666 | 0.000 | 1.340 | 1.070 | 0.799 | 27.4 | 952 | 37.4 |
| 0.477 | 0.392 | #NULL! | 1.673 | 1.711 | 1.800 | 1.830 | 1.989 | 0.775 | 0.771 | 0.000 | 0.750 | 0.720 | 0.960 | 25.0 | 945 | 42.9 |
| 0.278 | 0.205 | #NULL! | 0.891 | 0.914 | 0.955 | 0.994 | 1.058 | 0.463 | 0.478 | 1.000 | 1.590 | 0.830 | 0.522 | 23.0 | 954 | 15.3 |
| 0.392 | 0.415 | #NULL! | 1.636 | 1.681 | 1.755 | 1.801 | 2.035 | 0.571 | 0.580 | 0.000 | 1.340 | 0.840 | 0.627 | 23.3 | 955 | 13.9 |
| 0.345 | 0.171 | #NULL! | 0.901 | 0.940 | 1.008 | 1.073 | 1.075 | 0.490 | 0.498 | 0.000 | 1.340 | 1.730 | 1.291 | 25.6 | 958 | 14.8 |
| 0.359 | 0.102 | #NULL! | 0.746 | 0.780 | 0.866 | 0.938 | 1.254 | 0.525 | 0.541 | 0.000 | 1.330 | 0.930 | 0.699 | 21.5 | 941 | 16.8 |
| 0.376 | 0.201 | #NULL! | 1.222 | 1.248 | 1.286 | 1.337 | 1.486 | 0.679 | 0.690 | 1.000 | 1.160 | 1.050 | 0.905 | 23.8 | 955 | 22.4 |
| 0.295 | 0.158 | #NULL! | 0.600 | 0.621 | 0.664 | 0.704 | 0.847 | 0.443 | 0.458 | 0.000 | 1.380 | 1.400 | 1.014 | 24.6 | 955 | 20.5 |
| 0.551 | 0.808 | #NULL! | 2.699 | 2.729 | 2.816 | 2.745 | 2.954 | 0.924 | 0.927 | 1.000 | 1.160 | 0.700 | 0.603 | 26.2 | 958 | 26.6 |
| 0.397 | 0.632 | #NULL! | 2.231 | 2.295 | 2.377 | 2.434 | 2.832 | 0.684 | 0.690 | 0.000 | 1.020 | 0.500 | 0.490 | 23.8 | 960 | 19.8 |
| 0.767 | 0.492 | #NULL! | 1.928 | 1.945 | 2.018 | 2.076 | 1.541 | 1.027 | 1.032 | 0.000 | #NULL! | #NULL! | #NULL! | #NULL! | #NULL! | #NULL! |
| 0.491 | 0.205 | #NULL! | 1.559 | 1.583 | 1.641 | 1.666 | 1.306 | 0.775 | 0.780 | 1.000 | 0.960 | 0.080 | 0.083 | 25.5 | 943 | 20.8 |
| 0.461 | 0.307 | #NULL! | 1.428 | 1.426 | 1.412 | 1.396 | 1.394 | 0.544 | 0.546 | 0.000 | 1.320 | 1.300 | 0.985 | 26.6 | 944 | 38.4 |
| 1.309 | 0.407 | #NULL! | 3.792 | 4.083 | 4.745 | 5.279 | 6.799 | 2.105 | 2.298 | 0.000 | 1.240 | 1.310 | 1.056 | 25.2 | 954 | 45.5 |
| 0.295 | 0.206 | #NULL! | 0.761 | 0.802 | 0.870 | 0.911 | 0.996 | 0.376 | 0.386 | 1.000 | 1.170 | 2.370 | 2.026 | 23.9 | 946 | 38.7 |
| 0.329 | 0.344 | #NULL! | 1.254 | 1.279 | 1.319 | 1.376 | 1.462 | 0.503 | 0.508 | 0.000 | #NULL! | #NULL! | #NULL! | #NULL! | #NULL! | #NULL! |
| 0.500 | 0.292 | #NULL! | 1.681 | 1.733 | 1.837 | 1.943 | 2.291 | 0.818 | 0.832 | 0.000 | 0.670 | 1.160 | 1.731 | 25.0 | 955 | 41.8 |
| 0.342 | 0.154 | #NULL! | 0.862 | 0.884 | 0.920 | 0.933 | 0.837 | 0.509 | 0.514 | 1.000 | 1.650 | 3.160 | 1.915 | 26.5 | 953 | 37.6 |
| 0.215 | 0.192 | #NULL! | 0.603 | 0.628 | 0.661 | 0.680 | 0.755 | 0.326 | 0.338 | 0.000 | 1.680 | 2.220 | 1.321 | 25.4 | 960 | 22.5 |
| 0.308 | 0.377 | #NULL! | 1.411 | 1.435 | 1.493 | 1.505 | 1.292 | 0.530 | 0.539 | 0.000 | 1.130 | 2.130 | 1.885 | 25.9 | 960 | 21.4 |
| 0.344 | 0.251 | #NULL! | 1.164 | 1.211 | 1.273 | 1.400 | 1.429 | 0.546 | 0.562 | 0.000 | 1.080 | 0.920 | 0.852 | 25.7 | 960 | 19.3 |
| 0.449 | 0.357 | #NULL! | 1.336 | 1.355 | 1.404 | 1.452 | 1.561 | 0.630 | 0.635 | 1.000 | 0.600 | 0.810 | 1.350 | 25.6 | 955 | 23.7 |
| 0.571 | 0.527 | #NULL! | 1.938 | 2.027 | 2.180 | 2.379 | 2.285 | 0.716 | 0.728 | 0.000 | 0.610 | 1.600 | 2.620 | 27.2 | 949 | 28.1 |
| 0.855 | 0.014 | #NULL! | 1.037 | 1.025 | 1.008 | 0.963 | 0.835 | 0.948 | 0.947 | 0.000 | 1.340 | 1.520 | 1.134 | 24.6 | 949 | 25.4 |
| 0.303 | 0.145 | #NULL! | 1.014 | 1.145 | 1.317 | 1.503 | 1.801 | 0.586 | 0.641 | 0.000 | 1.400 | 1.230 | 0.879 | 24.7 | 962 | 15.9 |
| 0.635 | 0.796 | #NULL! | 2.188 | 2.260 | 2.334 | 2.369 | 2.180 | 0.706 | 0.714 | 0.000 | 0.690 | 1.500 | 2.174 | 21.8 | 959 | 23.9 |
| 0.512 | 0.586 | #NULL! | 1.978 | 2.000 | 2.025 | 2.047 | 2.200 | 0.754 | 0.759 | 0.000 | #NULL! | #NULL! | #NULL! | #NULL! | #NULL! | #NULL! |
| 0.376 | 0.316 | #NULL! | 1.161 | 1.185 | 1.221 | 1.247 | 1.247 | 0.434 | 0.439 | 0.000 | 1.590 | 1.180 | 0.742 | 21.8 | 945 | 24.6 |
| 0.461 | 0.263 | #NULL! | 1.104 | 1.160 | 1.249 | 1.319 | 1.225 | 0.628 | 0.651 | 0.000 | 1.730 | 1.540 | 0.890 | 24.9 | 955 | 13.6 |
| 0.328 | 0.090 | #NULL! | 0.923 | 0.991 | 1.157 | 1.343 | 2.009 | 0.611 | 0.652 | 0.000 | 1.720 | 2.480 | 1.442 | 24.9 | 954 | 11.0 |
| 0.295 | 0.194 | #NULL! | 0.711 | 0.723 | 0.763 | 0.807 | 0.860 | 0.378 | 0.384 | 1.000 | 0.750 | 1.640 | 2.187 | 25.2 | 955 | 11.2 |
| 0.602 | 0.314 | #NULL! | 1.767 | 1.797 | 1.879 | 2.171 | 0.956 | 0.794 | 0.765 | 0.000 | 1.120 | 0.640 | 0.571 | 26.2 | 952 | 38.4 |
| 0.350 | 0.084 | #NULL! | 0.707 | 0.772 | 0.910 | 1.083 | 1.482 | 0.440 | 0.473 | 0.000 | 1.700 | 3.660 | 2.153 | 24.9 | 941 | 19.6 |
| 0.222 | 0.103 | #NULL! | 0.379 | 0.387 | 0.404 | 0.418 | 0.437 | 0.236 | 0.240 | 0.000 | 0.670 | 1.050 | 1.567 | 23.2 | 954 | 13.1 |
| 0.387 | 0.395 | #NULL! | 1.350 | 1.350 | 1.325 | 1.229 | 0.936 | 0.593 | 0.593 | 1.000 | 1.170 | 1.030 | 0.880 | 25.3 | 944 | 31.7 |
| 0.433 | 0.205 | #NULL! | 1.240 | 1.257 | 1.285 | 1.274 | 1.420 | 0.741 | 0.747 | 0.000 | 0.670 | 0.920 | 1.373 | 26.4 | 944 | 27.4 |
| 0.432 | 0.227 | #NULL! | 1.002 | 1.031 | 1.064 | 1.087 | 1.076 | 0.614 | 0.631 | 1.000 | 1.740 | 1.950 | 1.121 | 26.3 | 954 | 39.8 |
| 0.349 | 0.069 | #NULL! | 0.722 | 0.739 | 0.772 | 0.799 | 0.684 | 0.601 | 0.614 | 0.000 | 1.370 | 1.230 | 0.898 | 25.5 | 952 | 44.7 |
| 0.469 | 0.447 | #NULL! | 1.945 | 2.014 | 2.193 | 2.400 | 2.603 | 0.754 | 0.763 | 1.000 | 0.990 | 1.380 | 1.394 | 22.1 | 958 | 25.7 |
| 0.488 | 0.187 | #NULL! | 1.510 | 1.625 | 1.866 | 2.098 | 2.840 | 0.812 | 0.865 | 1.000 | 1.740 | 2.650 | 1.523 | 26.3 | 955 | 40.8 |
| 0.537 | -0.075 | #NULL! | 0.860 | 0.875 | 0.907 | 0.932 | 0.955 | 0.889 | 0.904 | 0.000 | 1.400 | 1.420 | 1.014 | 25.3 | 946 | 48.8 |
| 0.315 | 0.228 | #NULL! | 1.142 | 1.223 | 1.383 | 1.517 | 1.735 | 0.572 | 0.598 | 0.000 | 1.740 | 2.580 | 1.483 | 24.8 | 954 | 20.5 |
| 0.439 | 0.107 | #NULL! | 0.921 | 0.946 | 1.000 | 1.067 | 1.194 | 0.743 | 0.765 | 1.000 | 1.680 | 2.750 | 1.637 | 24.4 | 942 | 21.3 |
| 0.297 | 0.264 | #NULL! | 1.042 | 1.067 | 1.133 | 1.198 | 1.329 | 0.377 | 0.379 | 0.000 | 0.720 | 1.920 | 2.667 | 22.3 | 950 | 18.9 |
| 0.508 | 0.466 | #NULL! | 1.325 | 1.335 | 1.359 | 1.375 | 1.432 | 0.598 | 0.601 | 0.000 | 0.600 | 0.960 | 1.600 | 24.8 | 955 | 10.6 |
| 0.500 | 0.300 | #NULL! | 1.548 | 1.554 | 1.551 | 1.541 | 1.499 | 0.903 | 0.908 | 1.000 | 1.010 | 1.160 | 1.149 | 25.0 | 960 | 19.2 |
| 0.290 | 0.186 | #NULL! | 0.740 | 0.733 | 0.730 | 0.727 | 0.691 | 0.397 | 0.395 | 0.000 | 0.770 | 1.520 | 1.974 | 26.4 | 946 | 31.7 |
| 0.427 | 0.048 | #NULL! | 0.939 | 0.987 | 1.115 | 1.323 | 1.206 | 0.647 | 0.664 | 1.000 | 1.100 | 1.900 | 1.727 | 24.1 | 954 | 12.8 |
| 0.260 | 0.094 | #NULL! | 0.530 | 0.534 | 0.539 | 0.539 | 0.534 | 0.365 | 0.369 | 1.000 | 0.660 | 1.380 | 2.091 | 25.6 | 959 | 16.3 |
| 0.515 | 0.292 | #NULL! | 1.712 | 1.797 | 1.899 | 1.989 | 1.936 | 0.809 | 0.827 | 1.000 | 0.670 | 0.230 | 0.343 | 25.4 | 944 | 22.2 |
| 0.526 | 0.254 | #NULL! | 1.635 | 1.677 | 1.732 | 1.781 | 1.941 | 0.974 | 0.993 | 0.000 | 1.050 | 1.860 | 1.771 | 26.4 | 948 | 30.9 |
| 0.285 | 0.128 | #NULL! | 0.764 | 0.789 | 0.835 | 0.878 | 1.097 | 0.408 | 0.418 | 1.000 | 1.090 | 1.650 | 1.514 | 27.2 | 955 | 35.6 |
| 0.780 | 0.071 | #NULL! | 1.327 | 1.349 | 1.393 | 1.421 | 1.468 | 0.929 | 0.939 | 0.000 | 0.820 | 0.900 | 1.098 | 28.1 | 951 | 35.6 |
| 0.273 | 0.250 | #NULL! | 1.160 | 1.255 | 1.416 | 1.681 | 2.060 | 0.540 | 0.578 | 0.000 | 0.790 | 1.710 | 2.165 | 26.6 | 944 | 26.6 |
| 0.525 | 0.113 | #NULL! | 0.537 | 0.537 | 0.505 | 0.471 | 0.454 | 0.527 | 0.538 | 0.000 | 1.660 | 1.910 | 1.151 | 24.7 | 949 | 25.5 |
| 0.435 | 0.087 | #NULL! | 0.729 | 0.781 | 0.906 | 1.050 | 1.234 | 0.532 | 0.563 | 0.000 | 1.640 | 2.040 | 1.244 | 24.4 | 941 | 21.3 |
| 0.380 | 0.348 | #NULL! | 1.389 | 1.449 | 1.535 | 1.599 | 1.690 | 0.608 | 0.625 | 1.000 | 1.550 | 1.950 | 1.258 | 24.4 | 956 | 12.1 |
| 0.357 | 0.273 | #NULL! | 1.203 | 1.249 | 1.350 | 1.406 | 1.441 | 0.562 | 0.578 | 0.000 | 1.630 | 2.320 | 1.423 | 25.0 | 963 | 17.2 |
| 0.297 | 0.462 | #NULL! | 1.182 | 1.198 | 1.246 | 1.294 | 1.630 | 0.484 | 0.489 | 0.000 | 1.260 | 0.750 | 0.595 | 24.9 | 958 | 21.8 |
| 0.430 | 0.618 | #NULL! | 1.535 | 1.553 | 1.643 | 1.730 | 1.826 | 0.562 | 0.563 | 0.000 | 1.020 | 2.070 | 2.029 | 25.5 | 957 | 14.9 |
| 0.493 | 0.306 | #NULL! | 1.350 | 1.354 | 1.381 | 1.415 | 1.504 | 0.560 | 0.556 | 1.000 | 1.150 | 1.990 | 1.730 | 21.1 | 942 | 52.1 |
| 0.240 | 0.127 | #NULL! | 0.658 | 0.701 | 0.763 | 0.826 | 1.013 | 0.380 | 0.397 | 1.000 | 1.140 | 1.090 | 0.956 | 25.0 | 954 | 12.8 |
| 0.404 | 0.345 | #NULL! | 0.921 | 0.921 | 0.966 | 1.005 | 1.080 | 0.630 | 0.630 | 1.000 | 1.450 | 3.140 | 2.166 | 26.1 | 954 | 27.1 |
| 0.598 | 0.557 | #NULL! | 2.111 | 2.122 | 2.120 | 2.113 | 2.560 | 0.801 | 0.804 | 0.000 | 1.260 | 1.650 | 1.310 | 26.6 | 953 | 37.8 |
| 0.367 | 0.262 | #NULL! | 1.134 | 1.189 | 1.263 | 1.324 | 1.446 | 0.564 | 0.579 | 0.000 | 1.640 | 3.100 | 1.890 | 24.7 | 964 | 20.1 |
| 0.419 | 0.184 | #NULL! | 1.161 | 1.245 | 1.386 | 1.460 | 1.634 | 0.596 | 0.623 | 1.000 | 1.000 | 1.300 | 1.300 | 23.8 | 962 | 17.4 |
| 0.384 | -0.048 | #NULL! | 0.469 | 0.480 | 0.502 | 0.527 | 0.551 | 0.522 | 0.537 | 0.000 | 1.470 | 1.780 | 1.211 | 24.7 | 954 | 10.2 |
| 0.372 | 0.253 | #NULL! | 1.190 | 1.235 | 1.312 | 1.377 | 1.555 | 0.637 | 0.661 | 1.000 | 1.110 | 1.400 | 1.261 | 24.4 | 948 | 15.9 |
| 0.286 | 0.400 | #NULL! | 1.158 | 1.193 | 1.261 | 1.356 | 1.326 | 0.390 | 0.395 | 0.000 | #NULL! | #NULL! | #NULL! | #NULL! | #NULL! | #NULL! |
